# Autonomous Hydrogel Actuators Programmed by Endogenous Biochemical Logic for Dual‐Stage Morphing and Drug Release

**DOI:** 10.1002/adma.202516809

**Published:** 2026-01-21

**Authors:** Yuchen Liu, Harischandra Potthuri, Alejandro Sosnik, Luai R. Khoury

**Affiliations:** ^1^ Department of Materials Science and Engineering Technion Israel Institute of Technology Haifa Israel

**Keywords:** autonomous actuators, biochemical logic, endogenous biochemical cues, hydrogels, protein‐driven materials

## Abstract

Designing soft materials that autonomously respond to complex physiological environments remains a fundamental challenge in biomedical systems engineering. Here, we report on a 3D‐printed hybrid protein‐polymer hydrogel actuator that operates via *endogenous biochemical logic*, enabling fully autonomous dual‐stage shape morphing and enzyme‐triggered drug release in gastric‐mimicking environments. The actuator comprises a bilayer structure: an active layer based on bovine serum albumin‐poly (ethylene glycol) diacrylate (BSA‐PEGDA), and a passive PEGDA layer. In acidic gastric fluid, the BSA‐PEGDA layer undergoes rapid conformational swelling, followed by delayed softening from pepsin‐mediated degradation, autonomously driving reversible shape transitions without manual intervention. By embedding doxorubicin (DOX) within the BSA‐PEGDA hydrogel network, the system achieves site‐specific, enzyme‐gated drug release that is tunable using pepstatin A as a biochemical inhibitor. High‐resolution digital light processing (DLP) printing enables the fabrication of complex autonomous actuators and microneedle‐equipped grippers capable of mucosal adhesion, catch‐and‐release behavior, and controlled delivery. This work establishes a materials design strategy where biochemical cues are used as programmable inputs to drive mechanical and therapeutic outputs, offering a robust platform for bioresponsive soft robotics and in situ drug delivery.

## Graphical abstract

A 3D‐printed BSA–PEGDA bilayer actuator performs biochemical logic, bending autonomously in acid and releasing its drug payload only when both acid and pepsin are present. This dual‐stage, enzyme‐gated mechanism enables autonomous catch‐and‐release motion and controlled gastric drug delivery, representing a programmable soft material powered by endogenous biochemical cues.

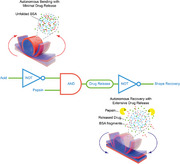

## 1 Introduction

Soft actuators, due to their responsiveness, flexibility, and adaptability, can safely interact with biological entities in complex dynamic environments [1]. This property endows them with broad application potential in biomedical fields, such as soft robotics, drug delivery systems, and tissue regeneration [2, 3, 4, 5]. For decades, biological motions—such as the bending, twisting, and folding behaviors of insects and vertebrates—have served as vital bioinspiration for actuator design [6]. Although current systems can undergo multi‐stage shape morphing in response to external stimuli like light encoding [7, 8], temperature [9, 10], electric fields [11, 12], fluid‐phase triggers [13, 14] and magnetic forces [15, 16, 17], they still heavily rely on manual intervention and remain limited in mimicking the complex, autonomous, and multi‐stage motion behaviors observed in living organisms to accomplish specific tasks [18]. For example, bee stinging involves an autonomous, multi‐stage process—precision positioning, active penetration, toxin injection, and rapid detachment—demonstrating autonomous, coordinated deformation. To enhance autonomy, temporal programming strategies have been developed to regulate deformation through preset time sequences. For instance, dynamic dual‐network hydrogels can adjust recovery rates [19], but deformation occurs immediately upon removal of the programming force, lacking initiation control. To address this, researchers have introduced light [20] or temperature [21] for pre‐programming, significantly improving deformation controllability. To further eliminate the need for manual intervention, including fuel‐driven feedback systems [22, 23], thiol‐based self‐catalytic reaction networks [24], pneumatic soft robot system [25], autonomous snapping and jumping polymer gels [26] and contact‐destructive hydrogel actuators [27] have successfully achieved autonomous multi‐stage shape morphing. However, their potential for in vivo applications remains uncertain.

Regarding the choice of materials for soft actuators, responsive polymers—particularly hydrogels—are considered ideal candidates, as they exhibit soft, water‐rich nature, excellent biocompatibility, porous structures [28], and sensitivity to multiple stimuli, such as pH, temperature, light, magnetic fields and ultrasound [29, 30]. Encouragingly, the dynamic adaptability of stimuli‐responsive polymers has facilitated their integration into soft robotics for drug delivery applications [31]. They have been successfully utilized in therapeutic grippers [32, 33], swelling‐enabled hydrogel actuators [34, 35], self‐degrading gastric retention devices [36, 37], a retractable needle system for gastric injections [38], and even a six‐armed star‐shaped drug delivery platform currently undergoing Phase III clinical trials [39, 40]. These devices significantly prolong drug release and enhance the precision of the delivery rate and body site.

Meanwhile, the design and synthesis of the stimuli‐responsive hydrogel materials themselves is also a crucial factor. Hybrid protein‐polymer hydrogels have recently garnered significant attention due to their inherent biocompatibility, and biological responsiveness [41, 42, 43]. These materials not only retain the shape‐morphing behavior derived from protein conformational changes under diverse endogenous stimuli—such as acids [44, 45], enzymes [46, 47, 48, 49, 50, 51], mechanical forces [52, 53], and metal ions [13, 54]—but also offer the mechanical stability of synthetic polymers, making them a preferred choice for developing in vivo actuators. However, these systems remain limited to single‐stage or non‐autonomous reversible shape transformations. Notably, their unique responsiveness to specific enzymes offers an effective strategy to overcome the inherent limitations of conventional stimulus in physiological environments—such as the risk of tissue damage or the poor tissue penetration of external stimulus signals. Enzymes, in particular, are increasingly recognized as ideal triggers due to their high efficiency, mild operating conditions, and strong specificity [55].

Among the various fabrication strategies for soft actuators, 3D printing offers a powerful approach for constructing hydrogel‐based actuators, enabling precise spatial arrangement of materials and enhancing both actuation controllability and functional integration [56, 57]. Therefore, developing a hybrid protein‐ polymer hydrogel actuator that enables autonomous, multi‐stage shape morphing, with 3D printability, biocompatibility, and controlled drug release, represents a valuable innovation for advancing intelligent biomedical devices and gastrointestinal drug delivery systems.

**FIGURE 1:**
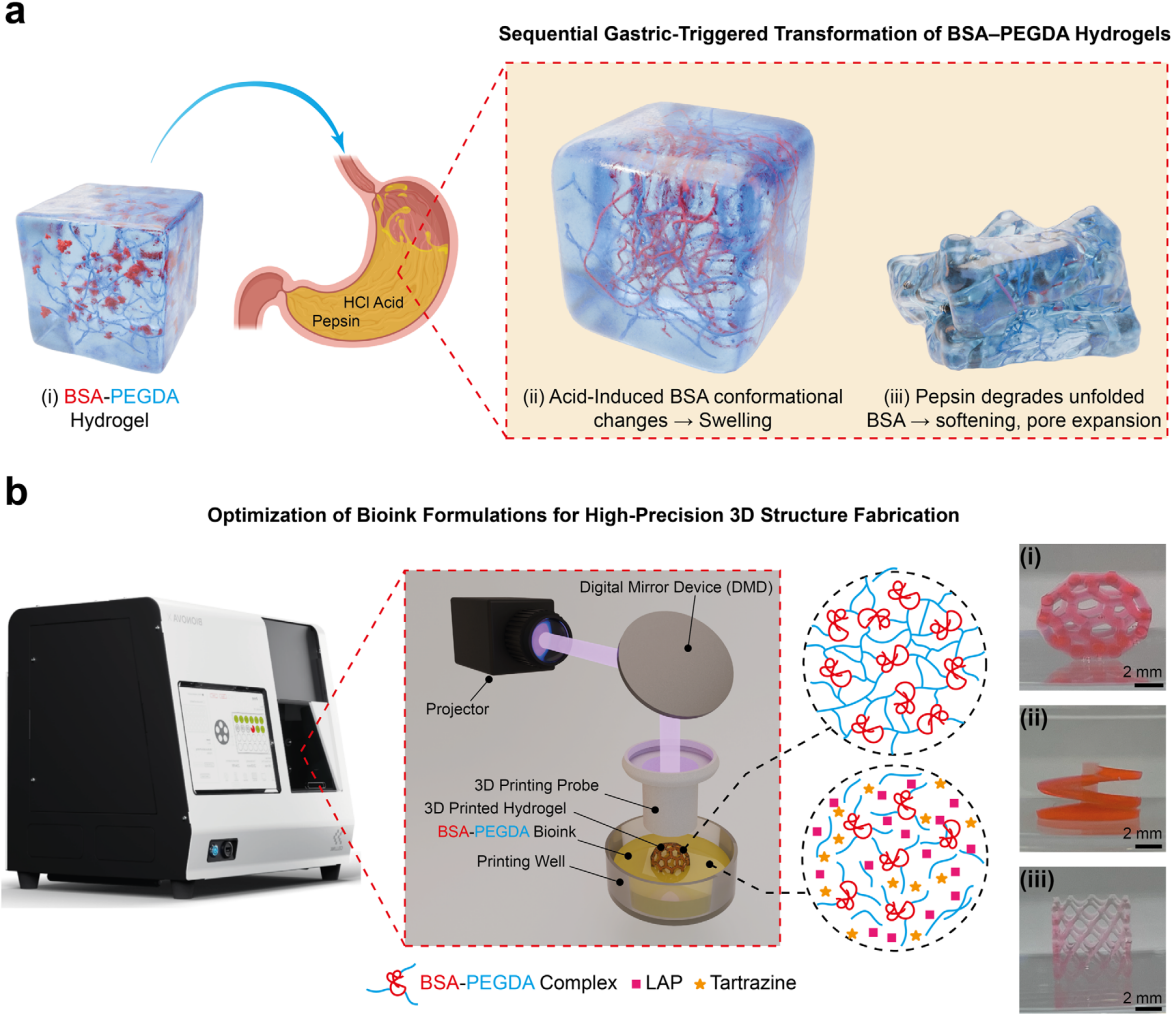
Acid‐ and enzyme‐triggered transitions of BSA‐PEGDA hydrogels and high‐fidelity 3D printing using an optimized BSA‐PEGDA bioink. (a) Schematic illustration of the responsive mechanism of the BSA‐PEGDA hydrogel in gastric fluid. (i) BSA molecules are covalently embedded within the PEGDA network, forming a single hybrid structure. In a neutral environment, BSA (red) maintains its globular conformation within the hydrogel. (ii–iii) Under gastric conditions, the hydrogel undergoes two sequential morphological transitions. In the early stage, acidic pH induces partial unfolding of BSA (depicted as red linear chains), while pepsin‐mediated degradation remains minimal, resulting in initial swelling. As time progresses, the degradative effect of pepsin gradually becomes dominant, leading to the degradation of more and more BSA, progressive hydrogel softening, and pore expansion, as evidenced by the disappearance of red BSA components and partial network collapse. (b) Synthesis and 3D printing of BSA‐PEGDA hydrogels. The BSA‐PEGDA complex was synthesized via an aza‐Michael addition reaction, in which the primary amine groups of lysine residues in BSA react with the acrylate moieties of PEGDA to form stable covalent bonds. This BSA‐PEGDA conjugate mixture was combined with LAP and tartrazine to produce a photocurable bioink. The ink was then loaded into a BIONOVA X (CELLINK) DLP 3D bioprinter equipped with a 405 nm violet light source (full width at half maximum ± 7.5 nm). Printing was performed at a light intensity of approximately 12 mW cm^−^^2^ (corresponding to 75% of the printer's adjustable range of 4–16 mW cm^−^^2^), with a layer thickness of 50 µm, XY resolution of 10 µm, Z precision of 4 µm (motor‐driven), and an exposure time of 10 s per layer. Under these conditions, 405 nm irradiation efficiently activates LAP to initiate free‐radical–mediated polymerization of PEGDA vinyl groups, enabling the layer‐by‐layer construction of stable hydrogel architectures with high fidelity. Following printing, samples were thoroughly rinsed in TRIS (10 min × 3 cycles) to remove unreacted monomers and residual photoinitiator. (i–iii) Illustrate well‐defined hollow C60, stent, and spiral structures fabricated via 3D printing using the optimized BSA‐PEGDA bioink formulation (BSA‐PEGDA: LAP: Tartrazine = 24:6:1, v/v/v). The constructs exhibit precise morphological definition and high structural fidelity, highlighting the critical role of tartrazine in enhancing the resolution of complex geometries.

Herein, we engineered a gastric fluid responsive hydrogel inspired by the acid‐induced structural deformation [58] and enzymatic degradation of globular proteins (Figure 1a). Specifically, we designed a bioink composed of bovine serum albumin‐poly (ethylene glycol) diacrylate (BSA‐PEGDA) and employed multi‐layered digital light processing (DLP) 3D printing to achieve high‐resolution fabrication for actuator construction. The optimized bioink formulation enabled the precise production of complex 3D objects (Figure 1b) and bilayer structures which integrating a stimuli‐responsive BSA‐PEGDA layer with an inert PEGDA counterpart. By harnessing gastric fluid—a physiologically relevant environment with low pH ∼2 and high pepsin activity (0.5–1 mg/mL) in the human body [59, 60]—as an endogenous stimulus, the actuator underwent programmable and autonomous transition from a 2D to a 3D structure, with subsequent recovery to its initial flat state, driven by sequential physical morphological transitions in the BSA‐PEGDA hydrogel (Figure 1a). Initially, acidic conditions triggered BSA structural deformation, resulting in rapid BSA‐PEGDA layer swelling; subsequently, pepsin‐mediated degradation gradually induced matrix collapse, leading to softening and increased porosity. During this process, the swelling and stiffness mismatch between the active BSA‐PEGDA layer and the inert PEGDA layer enabled autonomous shape morphing, with the PEGDA layer providing both bending resistance and elastic restoring force for shape recovery. Furthermore, the BSA facilitated the non‐covalent incorporation of the anticancer drug doxorubicin (DOX), forming a BSA‐PEGDA‐DOX bioink. The interaction between DOX and BSA was primarily governed by electrostatic attraction, hydrogen bonding, and hydrophobic interactions. The resulting 3D‐printed gripper demonstrated autonomous catch‐and‐release of a ball or a mucus‐mimicking layer, along with enzyme‐responsive drug delivery in gastric‐mimicking environments, highlighting its potential as a soft robotic system for gastric therapy. We also found that the rates of both drug release and shape recovery could be finely tuned by adjusting the concentration of pepstatin A (PepA), a selective pepsin inhibitor. Moreover, the very good cell compatibility of BSA‐PEGDA and PEGDA hydrogels in vitro was demonstrated in the mucus‐secreting intestinal epithelial cell line HT29‐MTX over 7 days.

## 2 Results

### 2.1 Formulation and Optimization of a Photocurable BSA‐PEGDA Bioink for High‐Fidelity 3D Printing

BSA, a natural globular protein, has attracted significant attention across numerous application fields due to its abundant availability in blood, low cost, and conformational flexibility [61, 62]. Incorporating BSA molecules into synthetic polymer hydrogels not only markedly enhances their physical properties but also enables the imparting of specific functionalities through structural modulation of BSA [63, 64]. To further expand its functionality and applications, we synthesized a BSA‐PEGDA conjugate by an aza‐Michael addition reaction to facilitate the reaction between lysine residues on the BSA surface and the acrylate groups of PEGDA (M.W. 700 Da) [65]. The optimization of the BSA:PEGDA ratio in the conjugation reaction left enough unreacted acrylate groups for chemical cross‐linking [45]. This mixture was subsequently cross‐linked via photoinitiated free radical polymerization of the acrylate groups of PEGDA and fabricated into hydrogels using DLP 3D printing technology. Specifically, BSA‐PEGDA (2.5–125 mM) or PEGDA (250 mM) mixtures were prepared in tris‐aminomethane (TRIS) buffer (20 mm Tris, 150 mm NaCl, pH ∼7.4) and mixed with the photoinitiator lithium phenyl‐2,4,6‐trimethylbenzoylphosphinate (LAP, 150 mm) at a 4:1 volume ratio to form a photocurable bioink. To evaluate its printability, we utilized DLP‐based 3D printing in a layer‐by‐layer mode to fabricate BSA‐PEGDA (2‐100 mM) and PEGDA (200 mM) hydrogels. However, the results revealed that light scattering caused over‐curing in non‐target areas, compromising the precision of fine structure printing. To address this issue, we introduced the light absorber tartrazine into the bioink mixture to regulate light intensity, thereby reducing over‐curing in non‐target regions and improving printing precision [66] (Figure 1b). Subsequently, we assessed the impact of varying tartrazine concentrations on the printing quality of 3D complex printing shapes, such as a spider web shape by evaluating the clarity and integrity of the mesh, determining the optimal concentration. The optimized bioink formulation consisted of BSA‐PEGDA/LAP at a 30:1 volume ratio with 2% (w/v) tartrazine and PEGDA/LAP at a 20:1 volume ratio with 2% (w/v) tartrazine (see Figure S1a). To validate the optimization, we printed complex and geometrically challenging structures—such as hollow C60, stent and spiral designs—using bioinks with and without tartrazine. Hydrogels lacking tartrazine exhibited poor fidelity in replicating these intricate forms, while those formulated with the optimized tartrazine concentration successfully yielded well‐defined hollow C60, stent and spiral structures (Figure 1b‐i–iii; Figure S1b).

### 2.2 Acid‐ and Enzyme‐Driven Modulation of BSA‐PEGDA Hydrogel Properties in Simulated Gastric Fluid

**FIGURE 2:**
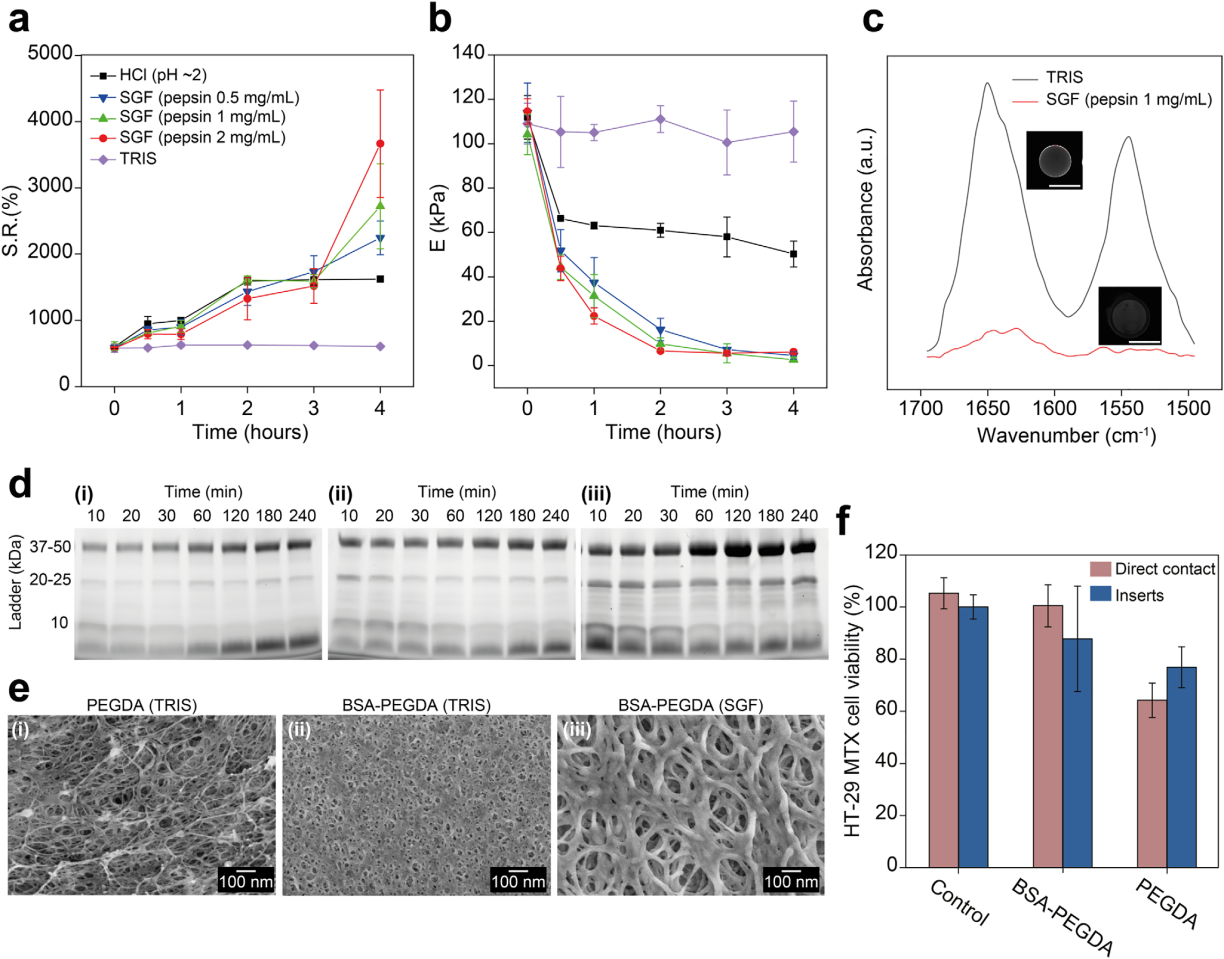
Swelling, degradation, structural analysis, and biocompatibility of BSA‐PEGDA hydrogels in gastric‐mimicking environments. (a) Swelling ratio (SR, %) of BSA‐PEGDA hydrogel at 37°C over 4 h in TRIS, HCl (pH ∼2), and SGFs (pepsin 0.5, 1, 2 mg/mL, pH ∼2). In HCl, SR increases for 2 h then stabilize. In SGF, SR rises significantly but less initially than in HCl (pH ∼2), as pepsin degrades BSA, reducing water absorption. Higher pepsin concentrations lower initial swelling but yield the highest SR after 4 h due to hydrogel erosion. In TRIS, SR remains stable. (b) Compressive Young's modulus (E, kPa) of BSA‐PEGDA hydrogel at 37°C over 4 h in TRIS, HCl (pH ∼2), and SGFs (pepsin 0.5, 1, 2 mg/mL, pH ∼2). At 30 min, Compressive Young's modulus drops significantly in SGF and HCl (pH ∼2) versus TRIS, with a slower decline in HCl (pH ∼2) than in SGF. In SGF, pepsin synergistically hasten stiffness loss, with higher pepsin levels accelerating the drop. Over time, the rate of stiffness declines in HCl (pH ∼2) slowed down, stabilizing at approximately 60 kPa, while the E in SGF falls to ∼10 kPa after 4 h. TRIS retains stable E values throughout the experiment. (c) ATR‐FTIR spectral analysis of BSA structure in BSA‐PEGDA hydrogels after 4 h of incubation in TRIS or SGF (pepsin 1 mg/mL, pH ∼2) at 37 °C. Compared to the TRIS group, amide I (∼1650 cm^−^^1^) and amide II (∼1550 cm^−^^1^) peaks decrease significantly, indicating BSA secondary structure disruption by enzymatic digestion. ANS serves as a hydrophobic fluorescent probe that associates with the hydrophobic regions of BSA, enabling sensitive detection of protein conformational changes and degradation. Inset ANS fluorescence images show reduced intensity with pepsin, suggesting less hydrophobic region exposure, further confirming BSA degradation in the hydrogel network. Scale bar: 8 mm. (d) SDS‐PAGE analysis was used to monitor BSA degradation and release from the hydrogel under SGF at 37 °C over 4 h. The increasing intensity of the primary BSA bands (37–50 kDa and <10 kDa) over time indicates progressive enzymatic digestion and peptide release from the hydrogel matrix. (e) Cryo‐SEM of PEGDA (200 mM) and BSA‐PEGDA (2–100 mM) hydrogels in TRIS versus treated BSA‐PEGDA in SGF (pepsin 1 mg/mL, pH ∼2) at 37°C for 4 h. PEGDA (TRIS) exhibits a disordered, large‐pored structure; BSA‐PEGDA (TRIS) shows uniform porosity. In SGF, BSA‐PEGDA degrades, displaying larger pores and a looser, partially broken network. (f) Cytocompatibility evaluation of BSA‐PEGDA (2–100 mM) and PEGDA (200 mM) hydrogels using the mucus‐secreting human intestinal epithelial cell line HT29‐MTX after 7 days of culture under direct and indirect (semipermeable inserts) contact conditions. Cell viability was quantified using the MTT assay. Both BSA‐PEGDA and PEGDA hydrogels exhibited high cytocompatibility, maintaining cell viabilities above 80% and around 70%, respectively, confirming their very good biocompatibility (Figure S3). All measurements were performed with *n* = 3 independent samples, and data are presented as mean ± SD.

We previously showed that the presence and conformational changes of BSA can regulate the physical properties of BSA‐PEGDA hydrogels in homogeneous solutions [45, 48]. Here, we propose controlling the swelling ratio and stiffness of hydrogels in a heterogeneous simulated gastric fluid (SGF) environment by modulating BSA structure and content. 3D‐Printed cylindrical BSA‐PEGDA hydrogels were incubated at 37°C and immersed in 3 mL of TRIS, HCl (pH ∼2), or SGFs (pepsin 0.5, 1, 2 mg/mL, pH ∼2), with swelling behavior characterized at 0, 0.5, 1, 2, 3, and 4 h. The findings demonstrated that the swelling ratio of BSA‐PEGDA hydrogels significantly increases in HCl (pH ∼2) and SGFs, while remaining stable in TRIS. Specifically, under acidic conditions (pH ∼2), BSA denaturation and structure deformation enhance water absorption and cause rapid volume expansion [67], with swelling reaching equilibrium after 2 h; in SGFs, the swelling ratio exhibits a sustained increase, with rapid volume expansion within 20 min. However, in the early stage, the swelling increment decreases with increasing pepsin concentration, as high concentrations of pepsin rapidly degrade BSA, reducing the protein content and disrupting the 3D hydrogel network (Figure 1a). This not only reduces the swelling capacity driven by the nanomechanical tension generated from acid‐induced BSA structural changes, but also weakens the elastic retraction of polymer chains, compromising the mechanical integrity needed for water retention and swelling [68]. These findings highlight the critical role of BSA in the swelling process and predict that sufficiently high pepsin concentration or activity would markedly suppress volume expansion in the early stage. Conversely, at later stages, higher pepsin concentrations result in a larger swelling ratio due to severe network degradation reducing dry mass, thereby elevating the calculated swelling ratio. In contrast, TRIS induces no notable BSA denaturation or degradation, maintaining a constant swelling ratio (Figure 2a). Overall, the swelling behavior of hydrogels in SGF is synergistically regulated by acid‐induced BSA structure deformation and pepsin degradation.

To further investigate the effect of SGF on the mechanical properties of the hydrogels, we evaluated the stiffness changes through compressive testing under different conditions. 3D‐Printed cylindrical BSA‐PEGDA hydrogels were immersed in 3 mL of TRIS, HCl (pH ∼2), or SGFs (pepsin 0.5, 1, 2 mg/mL, pH ∼2), incubated at 37°C for 4 h, and measured at 0, 0.5, 1, 2, 3, and 4 h. It was observed that stiffness significantly decreases in acidic conditions and SGFs compared to the TRIS group. In the TRIS group, stiffness stabilizes at approximately 110 kPa after 4 h, showing no significant change, indicating that this condition does not affect stiffness. In the HCl (pH ∼2) group, the stiffness decreased from 110 to 65 kPa within the first 30 min, primarily due to BSA structural deformation, which increases chain flexibility and loosens the cross‐linked network, resulting in reduced stiffness. This relaxation facilitates water absorption, leading to hydrogel swelling that further increases the spacing between polymer chains, decreases network density, and compromises mechanical strength. The rate of stiffness decline slowed down thereafter, likely due to the hydrogel reaching a structural equilibrium that limited further softening. In SGFs, stiffness reduction accelerates with increasing pepsin concentrations, surpassing the HCl group within 30 min, suggesting that acid and enzyme synergistically induce softening by disrupting the structural integrity of the BSA‐PEGDA hydrogel network; over time, the stiffness across all groups in SGFs converged, approaching ∼10 kPa. (Figure 2b). These swelling and stiffness changes reveal that, in SGF, early acidic conditions induce BSA structural deformation, loosening the network and manifesting as rapid volume expansion; later, intensified pepsin degradation further disrupts and degrades the cross‐linked structure, significantly reducing stiffness and softening the hydrogel. This process elucidates the time‐dependent dynamic changes in the physical properties of BSA‐PEGDA hydrogels under physiological gastric conditions.

To elucidate the molecular mechanisms underlying the aforementioned changes in the physical properties, we employed attenuated total reflectance‐Fourier Transform‐Infrared Spectroscopy (ATR‐FTIR) and 8‐anilino‐1‐naphthalenesulfonic acid (ANS) fluorescence probes to monitor conformational changes of BSA within BSA‐PEGDA hydrogels, while using Sodium Dodecyl Sulfate‐Polyacrylamide Gel Electrophoresis (SDS‐PAGE) to assess BSA degradation and peptide release. Specifically, we compared ATR‐FTIR spectra in the 1500–1700 cm^−^^1^ region–encompassing the protein amide I (1600–1700 cm^−^^1^) and amide II (1500–1600 cm^−^^1^) characteristic vibrational peak—after incubating the hydrogel in TRIS and SGF (pepsin 1 mg/mL, pH ∼2) at 37°C for 4 h, with changes in peak shape and position reflecting BSA conformational adjustments and degradation under SGF. Concurrently, ANS fluorescence probes were used to further validate the effects of SGF on BSA structure. As a hydrophobic probe, ANS binds to BSA hydrophobic groups, and its fluorescence intensity variations sensitively detect protein conformational changes or degradation. The results showed that, in TRIS, BSA retained its typical dual‐peak profile, whereas, after SGF treatment, these peaks markedly diminished or disappeared, indicating significant BSA degradation (Figure 2c). Moreover, a substantial reduction in ANS fluorescence intensity further confirmed that SGF (pepsin 1 mg/mL, pH ∼2) efficiently degrades BSA within 4 h at 37°C, leading to pronounced conformational changes and consequently affecting hydrogel structural integrity (Figure 2c). To further verify that these changes originate from enzymatic cleavage of BSA, SDS‐PAGE was also performed on pure BSA (2 mM) and BSA‐PEGDA (2–100 mM) solutions under the same experimental conditions. For the solution‐based BSA controls, in TRIS (37°C, 4 h), both samples exhibited no change in band intensity relative to their initial states, confirming their stability under non‐degradative conditions. Upon exposure to SGFs (pepsin 0.5, 1, 2 mg/mL, pH ∼2), however, free BSA was completely degraded. Compared with the SGF‐only control, both BSA and BSA‐PEGDA groups exhibited intensified bands after incubation, confirming that these additional bands originated from BSA degradation products. As expected, PEGDA hydrogels (in TRIS) showed no detectable protein bands, demonstrating that PEGDA itself does not contribute to the observed electrophoretic changes (Figures S2). For the hydrogel group, the results showed that in SGF (pepsin 0.5, 1, and 2 mg/mL, pH ∼2), the fragmentation profile closely matched that observed in the solution‐based BSA controls. However, the BSA fragments within the hydrogel samples increased gradually over time (10, 20, 30, 60, 120, 180, and 240 min). Unlike the solution phase, no instantaneous digestion occurred because the BSA molecules embedded within the hydrogel network were spatially confined, which reduced their exposure to pepsin and susceptibility to rapid enzymatic degradation. As enzymatic degradation of BSA continued and network pores expanded during softening, degraded fragments were gradually released, resulting in the progressive accumulation pattern observed within the gel. This finding suggests that pepsin‐mediated hydrolysis gradually degrades internal BSA and releases it into the external solution through a slow accumulation process (Figure 2d; Figures S2). Notably, in SGF (pepsin 2 mg/mL, pH ∼2), protein fragment density increased rapidly within 30 min, peaking after 2 h (Figure 2d‐iii), demonstrating that pepsin concentration significantly influences BSA degradation rates, with higher concentrations accelerating initial degradation. This phenomenon also indirectly indicates that the early suppression of hydrogel swelling by high concentrations of pepsin is primarily due to its degradation of BSA, leading to the release of protein components from the network into the external environment. Collectively, these findings confirm that the evolution of SDS‐PAGE band intensity results from pepsin‐mediated BSA cleavage and further support the proposed enzyme‐triggered softening mechanism.

To further visualize the impact of this degradation on hydrogel microstructure, we employed cryogenic‐scanning electron microscopy (cryo‐SEM) to examine microstructural changes in BSA‐PEGDA hydrogels before and after treatment in SGF. Imaging revealed that PEGDA (200 mM) hydrogels in TRIS exhibited significant pore size variability (approximately 10–100 nm) and a disordered network structure, reflecting the unevenness and uncontrollability of PEGDA cross‐linking, resulting in heterogeneous pore distribution and structural diversity (Figure 2e‐i). In contrast, BSA‐PEGDA (2–100 mM) hydrogels in TRIS displayed uniform pore sizes and a compact structure. Although direct comparison with PEGDA (200 mM) hydrogels is not feasible, existing studies suggest that low‐concentration PEGDA hydrogels typically exhibit larger pores and disorder [48], indicating that BSA plays a critical role in regulating hydrogel microstructure. This may be attributed to the introduction of BSA, which guides the ordered arrangement of PEGDA chains through covalent interactions, thereby significantly enhancing network uniformity and compactness. These findings also confirm that the 3D printing process does not substantially alter the structural properties of BSA‐PEGDA hydrogels (Figure 2e‐ii). After treatment with SGF (pepsin 1 mg/mL, pH ∼2), BSA‐PEGDA hydrogel pore sizes significantly increased, reaching up to 300 nm, due to pepsin degradation disrupting the cross‐linked network and eroding the internal structure, thereby enlarging pores (Figure 2e‐iii).

As these hydrogels are envisioned for in vivo biomedical applications, their biosafety was preliminarily evaluated in a model of the gastrointestinal epithelium in vitro. Cytocompatibility was assessed with the mucus‐secreting human intestinal epithelial cell line HT29‐MTX, originally established at the European Collection of Authenticated Cell Cultures (ECACC, Cat. No. 12040401) and obtained from Sigma‐Aldrich, and cultured following the established protocol that closely mimics the epithelial environment of the small intestine—the site where the gastric actuator and its degradation products would ultimately interact [69]. Cell viability was quantified using the 3‐[4,5‐dimethylthiazol‐2‐yl]‐2,5‐diphenyl tetrazolium bromide (MTT) assay, which measures mitochondrial metabolic activity as an indicator of proliferation and cytotoxicity. Both direct and indirect contact experimental setups were used to assess the effects of physical contact and diffusible species on cell response after 7 days of culture. In both setups, BSA‐PEGDA hydrogels maintained high cell viability (>80%), while PEGDA hydrogels showed slightly lower but still acceptable values (∼70%), confirming the very good intrinsic cell compatibility of both systems (Figure 2f; Figure S3). These results are expected to be even better in vivo owing to the presence of a relatively thick (50–450 µm) and uniform mucus layer that protects the underlying gastric epithelium from chemical, biological and mechanical insults [70].

### 2.3 Development and Characterization of Gastric‐Responsive BSA‐PEGDA Hydrogel Actuators

**FIGURE 3:**
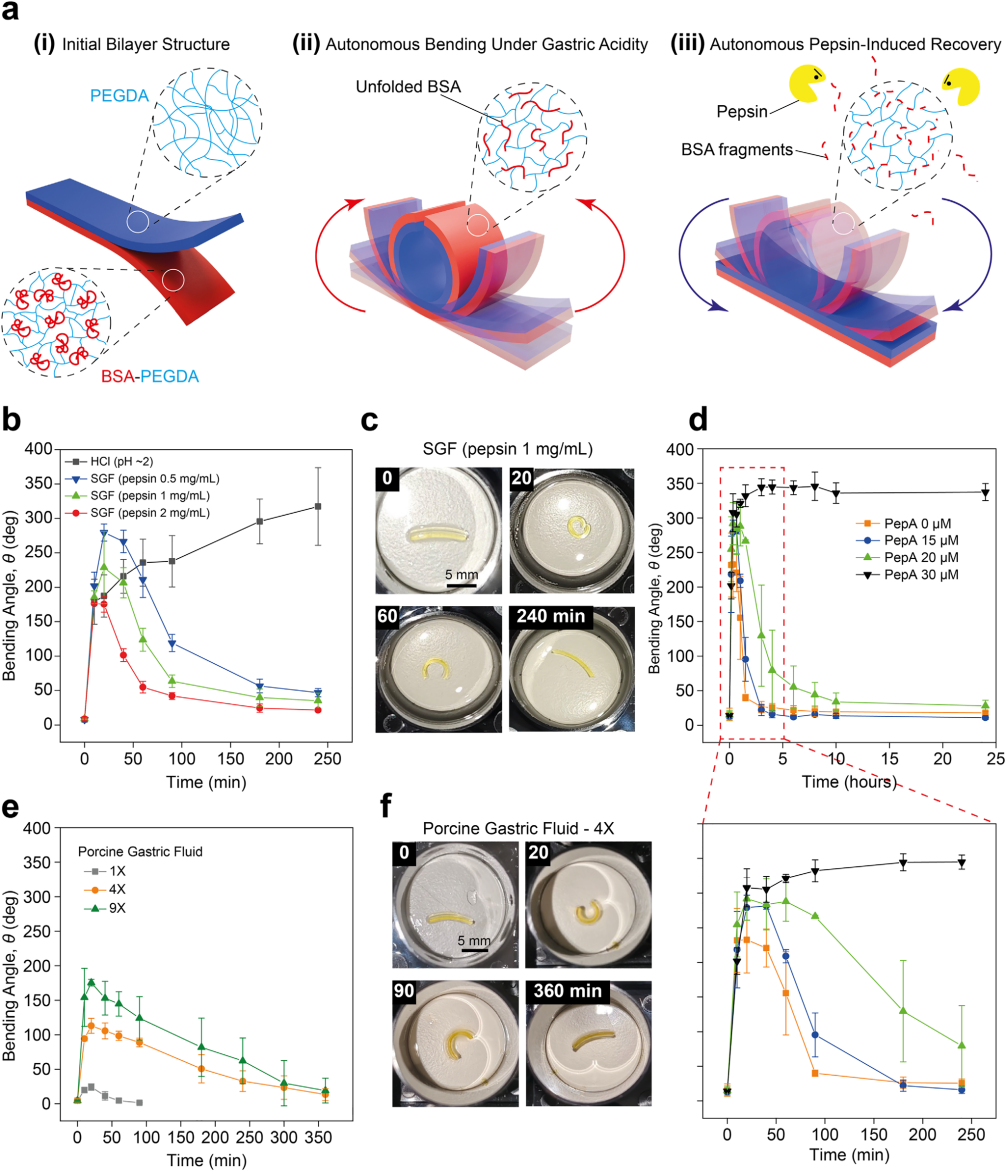
Sequential Shape Morphing of Bilayer Hydrogel Actuators under Acidic, Enzymatic, and Inhibitor‐Modulated Gastric Conditions. (a) Schematic of autonomous shape morphing of a 3D‐printed BSA‐PEGDA (2–100 mM)/PEGDA (200 mM) bilayer hydrogel actuator, covalently bonded (i), In SGF at 37 °C, initial bending occurs as structural deformation of BSA enhances water absorption, leading to swelling of the BSA‐PEGDA layer. (ii). Over time, enzymatic degradation of BSA gradually softens the BSA‐PEGDA layer, allowing the PEGDA layer to recover the original shape (iii). (b,c) Shape morphing response of hydrogels in HCl (pH ∼2) and SGFs (pepsin 0.5, 1, 2 mg/mL, pH ∼2). Both hydrogel actuators exhibited a rapid increase in bending angle within the first 20 min, with HCl (pH ∼2) treatment resulting in continuous bending, reaching approximately θ*_max_* ∼320° after 4 h. In contrast, in the presence of pepsin, the bending angle initially increased but gradually decreased, ultimately returning to a straight shape. The initial maximum bending angle in SGFs was lower at higher pepsin concentrations, likely due to BSA degradation reducing water absorption and lowering stiffness. (d) BSA‐PEGDA/PEGDA bilayer hydrogels were incubated at 37°C in 3 mL of SGF (pepsin 1 mg/mL, pH ∼2) with C*_PepA_* ∼0, 15, 20, and 30 µM. Initial maximum bending occurred within 20 min, with angles rising from θ*_max_* ∼240° (C*_PepA_∼*0 µM) to ∼300° (C*_PepA_∼*30 µm) as PepA concentration increased. The recovery time to a straight shape increased with inhibitor concentration: 120 min (C*_PepA_∼*0 µM), 180 min (C*_PepA_∼*15 µM), 600 min (C*_PepA_∼*20 µM), while the C*_PepA_∼*30 µM group remained bent (θ*_max_* ∼330°) even after 24 h. These findings demonstrate that PepA inhibits pepsin activity in a concentration‐dependent manner, slowing BSA‐PEGDA degradation and regulating the second‐stage shape morphing. (e,f) Shape morphing behavior in PGF with different dilution volume ratios (1X, 4X, 9X). In the 1X group, the hydrogel did not bend due to rapid BSA degradation by high‐concentration pepsin, which prevented sufficient swelling. In contrast, the 4X and 9X groups exhibited both bending and gradual recovery, with the bending angle inversely correlated with pepsin concentration. Additionally, the recovery phase in PGF was slower than in SGF, possibly due to impurities affecting pepsin activity. All measurements were performed with *n* = 3 independent samples, and data are presented as mean ± SD.

Leveraging the behavior of BSA‐PEGDA hydrogels in SGF‐rapid acid‐induced swelling followed by enzyme‐driven softening (Figure 1a), we developed an SGF‐responsive autonomous dual‐stage shape‐morphing actuator. To this end, we employed multi‐material BIONOVA X DLP 3D printing to fabricate a BSA‐PEGDA (2–100 mM)/ PEGDA (200 mM) bilayer hydrogel, with its interface covalently bonded to ensure interlayer stability, achieving a thickness of 0.3 mm for each layer (Figure 3a‐i). In SGF, the BSA‐PEGDA layer undergoes significant swelling due to BSA structural deformation, while the PEGDA layer remains relatively unchanged, serving as a chemically inert, highly cross‐linked, enzyme‐resistant, mechanically robust network that maintains stiffness and elasticity (Figure S6). Specifically, PEGDA possesses a poly(ethylene glycol) backbone (–CH_2–_CH_2–_O–) that lacks peptide bonds, making it resistant to pepsin degradation and chemically stable under strong acidic conditions [71, 72]. The bending of the bilayer actuator is driven by a swelling mismatch between the active BSA‐PEGDA layer and the inert PEGDA layer. This expansion is constrained by the PEGDA layer, generating interfacial stress that induces bending (Figure 3a‐ii). As pepsin progressively degrades the BSA chains, the hydrogel's 3D network becomes increasingly disrupted, resulting in a pronounced reduction in stiffness. Once the BSA‐PEGDA layer can no longer support the bent configuration, the elastic PEGDA layer drives the structure back to its original shape (Figure 3a‐iii). The results showed that the actuator's bending and recovery depend on pepsin concentration. At low pepsin levels (0.5 mg/mL), slower enzymatic degradation allows sufficient time for BSA structural deformation and hydrogel network expansion, generating significant swelling and a large bending angle (θ*_max_* ∼300° within 20 min; see Video S1). In contrast, higher pepsin concentrations (1 and 2 mg/mL) lead to rapid breakdown of the hydrogel network, preventing the buildup of nanomechanical tension and limiting swelling. This causes early mechanical collapse of the active layer and compromises its ability to maintain the bent configuration. As a result, smaller maximum bending angles are observed (θ*_max_* ∼230° and ∼180°, respectively). For the SGF groups containing 1 and 2 mg/mL pepsin, the curled rings were opened within 60 and 40 min, respectively, and recovered to an almost straight shape at approximately 120 and 90 min. In contrast, for the 0.5 mg/mL group, the ring gradually opened within 90 min but did not fully return to a straight shape within 4 h, retaining a bending angle of approximately 50°, and is expected to require a considerably longer time to recover fully. In HCl (pH ∼2), the absence of pepsin preserved the 3D structure of the BSA‐PEGDA network, allowing progressive bending to a maximum angle of θ*_max_* ∼320° (Figure 3b,c; Figure S4a).

Based on the variability in bilayer hydrogel shape morphing behavior induced by different pepsin concentrations, we hypothesized that the rate of second‐phase shape morphing could be modulated by regulating pepsin activity. Therefore, we employed the pepsin inhibitor, PepA, to control pepsin activity, influencing the degradation behavior of BSA‐PEGDA hydrogel, thereby modulating the recovery rate of the hydrogel actuator during shape morphing. The bilayer hydrogels were immersed in 3 mL of a mixed solution containing SGF (pepsin 1 mg/mL, pH ∼2) and varying concentrations of PepA (C*_PepA_∼*0, 15, 20, and 30 µM), incubated at 37°C, and bending angles during shape morphing were measured at different time points (0, 10, 20, 40 min, and 1, 1.5, 3, 4, 6, 8, 10 and 24 h). The observations showed that all samples completed initial bending within 20 min, with the maximum bending angles increasing from θ*_max_* ∼240° at C*_PepA_∼*0 µM to ∼300° at C*_PepA_∼*30 µM as PepA concentration increased. More notably, shape recovery time extended with increasing PepA concentration. The samples in C*_PepA_∼*0 µM opened from the ring shape within 60 min and recovered to a straight bilayer shape after 120 min, while those in C*_PepA_∼*15 and 20 µM opened within 90 and 180 min, respectively, and recovered after 180 and 600 min. In contrast, the samples in C*_PepA_∼*30 µM remained bent after 24 h (θ*_max_* ∼330°) (Figure 3d; see Figure S4b). This phenomenon indicates that PepA effectively inhibits pepsin activity in a concentration‐dependent manner, significantly slowing down the degradation extent of the BSA‐PEGDA layer, thereby preventing the actuator from softening and being driven back to its initial shape by the PEGDA layer, underscoring the critical role of pepsin activity in regulating the dual‐stage shape morphing of bilayer hydrogels.

Interestingly, we immersed the actuator in freshly collected ex vivo porcine gastric fluid (PGF) at 37°C to evaluate its autonomous shape morphing behavior and adaptability to complex environments. The real PGF used in these experiments was obtained from healthy adult pigs at a Rambam Medical Center slaughterhouse within 2 h postmortem. All procedures complied with animal protection regulations, and the pigs were not fasted prior to sample collection.

We hereby confirm that no ethics approval was required for the collection or use of porcine gastric fluid or porcine blood, as all materials were obtained postmortem and no procedures were performed on live animals.

Prior to use, the pH of the PGF was adjusted to ∼2 before use, and, given the unknown pepsin concentration, it was diluted in HCl (pH ∼2) at volume ratios of 1X, 4X, and 9X for testing. The findings revealed that in undiluted PGF (1X), the actuator exhibited minimal shape morphing, as high pepsin concentrations rapidly degraded BSA, suppressing acid‐induced volume expansion while the rapid stiffness reduction of the BSA‐PEGDA layer limited shape morphing. In contrast, at PGF (4X, and 9X) dilutions, the hydrogel displayed autonomous shape morphing behavior (Figure 3e,f; see Figure S4c), though the high pepsin concentration group showed a reduced maximum bending angle and a slower second‐phase shape morphing rate compared to lab‐prepared SGF, possibly due to complex biological components in PGF reducing or restricting pepsin activity over time, thereby slowing down the hydrogel's straightening process. These findings highlight the impact of enzyme activity fluctuations in *ex vivo* gastric fluid on shape morphing behavior.

### 2.4 Programmable Shape‐Morphing of Complex Hydrogel Architectures via Endogenous Gastric Cues

**FIGURE 4:**
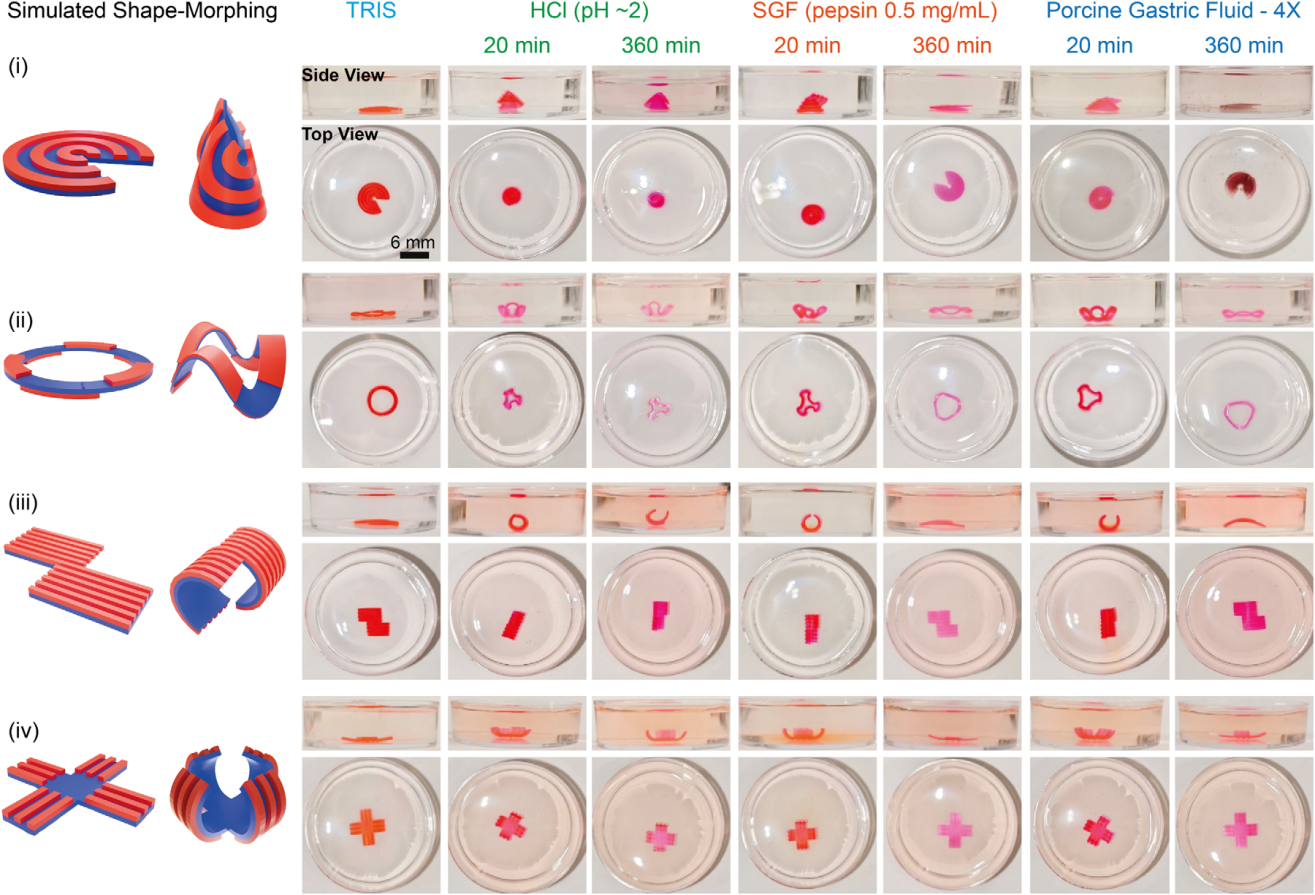
Gastric‐Cue–Driven Shape Morphing of Complex 3D‐Printed Bilayer Hydrogel Actuators. Four different 3D‐printed hydrogel actuator shapes (i‐pyramid, ii‐ring, iii‐bamboo, iv‐box) were evaluated for autonomous shape morphing in various environments at 37°C for 6 h, including HCl (pH ∼2), SGF (pepsin 0.5 mg/mL, pH ∼2), and PFG (4X, pH ∼2). In TRIS, the hydrogel maintained its original shape. In HCl (pH ∼2), the hydrogel transitioned from a flat shape to a stable 3D structure. In SGF or PGF, the hydrogel actuators initially bent from a flat structure to 3D structure before gradually recovering to its original shape.

By combining high‐precision 3D printing with an endogenous logic‐driven actuation mechanism –acid‐triggered swelling followed by enzyme‐mediated softening—a geometry–material coupling strategy was established to enable application‐oriented actuator design and prediction of shape‐morphing pathways [73]. Four representative actuator geometries were fabricated to demonstrate this concept. The pyramid actuator functioned as a self‐elevating platform, transforming from a planar structure into a pyramid under acidic conditions to lift a payload, followed by enzyme‐mediated softening that enabled autonomous unloading, completing a sequential lifting–holding–releasing cycle. The trilayer ring actuator served as a sampling or gentle clamping device, maintaining temporary fixation of soft tissues or payloads under peristaltic motion and releasing upon enzymatic degradation. The bamboo‐like actuator, printed as a planar sheet, autonomously rolled into a tubular configuration in acidic conditions and subsequently expanded under enzymatic action, functioning as a short‐term luminal scaffold for transient structural support. The box‐like actuator integrated multi‐arm gripping with planar lifting; upon acid exposure, its arms folded inward to secure or elevate a payload, maintaining stability during operation and releasing automatically through enzyme‐induced softening. The results revealed that these hydrogels achieved specific shape morphing along predetermined pathways in gastric fluid environments (pepsin 0.5 mg/mL, 4X diluted PGF, pH ∼2). Within 20 min, the pyramid‐shaped hydrogel (Figure 4i), due to the arc structure and non‐closed opening design of its BSA‐PEGDA surface layer, bent or rotated toward the opening edge, ultimately forming a pyramid‐like 3D structure; the trilayer ring structure (Figure 4ii), with a PEGDA middle layer and staggered BSA‐PEGDA bottom and top layers, drove directional bending in the bilayer regions, forming a wave‐surrounded 3D flower shape with area contraction and side‐folding elevation; meanwhile, bamboo‐like and box‐like hydrogels (Figure 4iii,iv), guided by linear BSA‐PEGDA strips on their surface layers, exhibited directional bending, with the bamboo‐shaped hydrogel gradually curling into a bamboo tube‐like structure and the box‐like hydrogel bending its four arms upward. As pepsin further degraded the BSA‐PEGDA layer, the layer stiffness decreased, and all shape‐morphing structures gradually returned to their initial state, demonstrating that these actuators can autonomously transition from flat patterns to specific 3D shapes through programmed control, and recover to flat upon completion of shape morphing in SGF or PGF, thus achieving autonomous‐bending and recovery (see Video S2). In contrast, in HCl (pH ∼2), the hydrogel actuators exhibited only the first‐stage bending response and maintained their 3D structure.

### 2.5 Biochemically Triggered Untethered Hydrogel Grippers for Autonomous Grasping and Release

**FIGURE 5:**
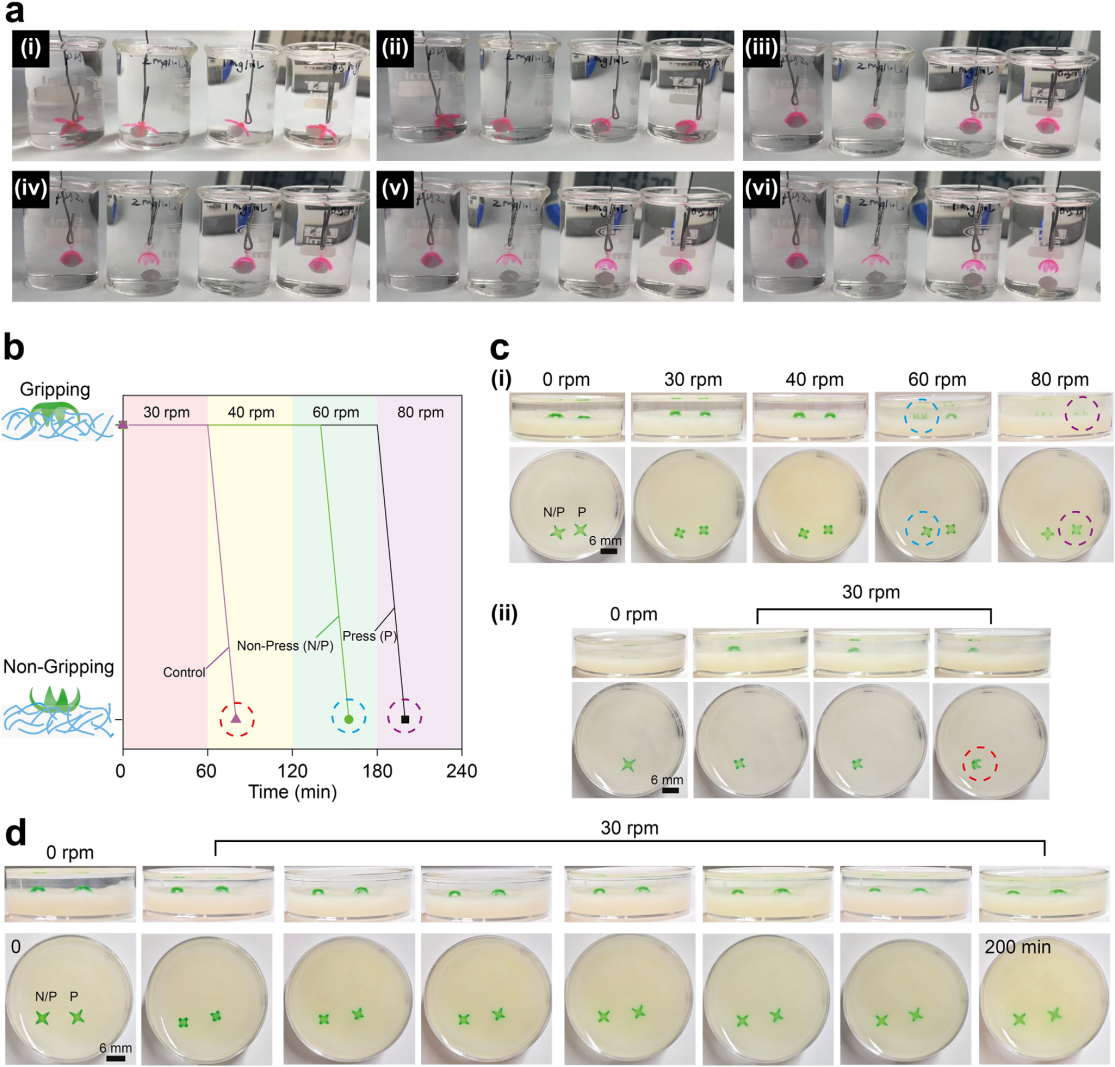
Autonomous Catch‐and‐Release Behavior of 3D‐Printed Hydrogel Grippers in Simulated Gastric and Mucus Environments. (a) Real‐time shape morphing of a 3D‐printed hydrogel gripper in HCl (pH ∼2) and SGFs (pepsin 2, 1, and 0.5 mg/mL, pH ∼2) (i‐vi), at 37°C. Over time, in SGF, the hydrogel gradually bent and gripped a plastic ball before releasing it and returning to its original shape. Higher pepsin concentrations accelerated hydrogel degradation, leading to earlier ball release, whereas lower concentrations resulted in slower release. In contrast, in HCl (pH ∼2), the hydrogel gripped the ball continuously without releasing it. (b,c) Gripping performance of left (N/P) and right (P) grippers under acidic and mucus‐like environments. (i) In HCl (pH ∼2), both grippers‐maintained grip at 30 and 40 rpm; the left‐side (N/P) gripper lost grip at 60 rpm, whereas the right‐side (P) gripper lost grip only at 80 rpm. (ii) In the control group, the gripper (non‐embedded) lost grip at 40 rpm after natural bending, indicating weak gripping performance. (d) In SGF (pepsin 1 mg/mL, pH ∼2) at 30 rpm, the gripper autonomously caught and released the simulated mucus layer.

Soft actuator‐based object manipulation plays a critical role in minimally invasive surgery and other biomedical applications; for example, untethered micro‐grippers have been employed for targeted drug delivery [33], active biopsy [74], and cell excision from tissue [75]. To leverage the autonomous shape morphing behavior of our stimuli‐responsive materials, we developed a four‐armed BSA‐PEGDA/PEGDA bilayer hydrogel gripper capable of manipulating and transporting payloads. The gripper was placed in HCl (pH ∼2) and SGFs (pepsin 0.5, 1, and 2 mg/mL, pH ∼2) to observe its shape morphing and grasping ability. It was observed that, the gripper tightly grasped a plastic ball (∼50 mg) and successfully lifted it within approximately 15 min; over time, the high‐concentration SGF group (pepsin 2 mg/mL, pH ∼2) released the ball first, followed by the 1 mg/mL and 0.5 mg/mL groups in sequence, while in HCl (pH ∼2), the gripper maintained its grasping state without release (Figure 5a; and see Video S3).

In addition to being used for grasping and placement tasks, another potential application for such devices is drug delivery. Inspired by therapeutic grippers [33] or mucoadhesive microneedle systems [76] capable of strong and prolonged fixation on the gastric mucosa, thereby improving drug retention and absorption. We designed a four‐armed microneedle gripper system with mucus‐penetrating capability and improved fixation stability on the gastric mucus layer. The gripper features sharp‐ended arms and consists of a three‐layered structure: the bottom layer is composed of BSA‐PEGDA (2–100 mM) hydrogel, the middle layer is 200 mM PEGDA hydrogel, and the top layer consists of rigid microneedles made from 25% (w/v) PEGDA (see Figure S4d). We systematically evaluated its penetration ability, gripping stability, and autonomous grasp‐and‐release behavior using a simulated mucus layer [77].

To verify whether the microneedle gripper can autonomously penetrate and grip the mucus layer in an HCl (pH ∼2) environment, we established three experimental groups: (1) pressing group, in which the gripper was placed on the mucus surface and briefly pressed to observe the gripping effect after microneedle embedding; (2) non‐pressing group, in which the gripper was placed directly on the mucus surface without applied force to evaluate its autonomous gripping and embedding capability; (3) control group, in which the gripper was placed in HCl (pH ∼2) without gripping the mucus layer to assess the natural stability in the absence of gripping. All experiments were conducted in a 37°C incubator with varying shaking speeds to simulate gastric motion.

Under HCl (pH ∼2) conditions, we gradually increased the shake speed (from 30 to 80 rpm, where each stage maintained for 1 h) to test the maximum gripping capability of the pressing (P) and non‐pressing (N/P) groups in the simulated mucus environment. In the experiment, the left‐side gripper represented the N/P group, which naturally gripped the mucus layer, while the right‐side gripper belonged to the P group, with microneedles embedded. The results showed that both grippers remained stably gripping at 30 and 40 rpm; at 60 rpm, the N/P group lost grip first, whereas the P group fully lost grip and flipped only at 80 rpm, indicating that microneedle embedding via pressing significantly enhanced gripping stability (Figure 5b,c‐i). In the control experiment, the gripper was first inverted (microneedles facing upward) and placed in HCl (pH ∼2) containing the mucus layer for 20 min to allow natural bending; it was then flipped and placed on the mucus surface, followed by a gradual increase in shake speed starting from 30 rpm to assess its standing stability under non‐embedded conditions. The results showed that the gripper gradually tilted during 1 h of shaking at 30 rpm and completely lost grip at 40 rpm, indicating weak gripping in the non‐embedded state (Figure 5b,c‐ii). These findings further confirm that, under shaking conditions, the microneedle gripper can autonomously penetrate and stably grip to the mucus layer, effectively improving its gripping performance. In SGF (pepsin 1 mg/mL, pH ∼2), we tested the P and N/P groups at a shaking speed of 30 rpm to simulate mild gastric peristalsis. During the experiment, the gripper was observed to autonomously bend from an initial state into a gripping state and then return to its original state, with no significant positional displacement (Figure 5d). This may be due to an increased contact area between the gripper and the surface after re‐expansion, which enhanced friction and suppressed slippage. Even under increased shaking speeds, no visible displacement was observed. This reversible grasp‐and‐release behavior is of great significance for therapeutic grippers that require autonomous gripping and release functionality in mucosal drug delivery.

### 2.6 DOX‐BSA Binding, Printability of DOX‐Loaded Bioinks, and Enzyme‐Regulated Drug Release from BSA‐PEGDA Hydrogels

**FIGURE 6:**
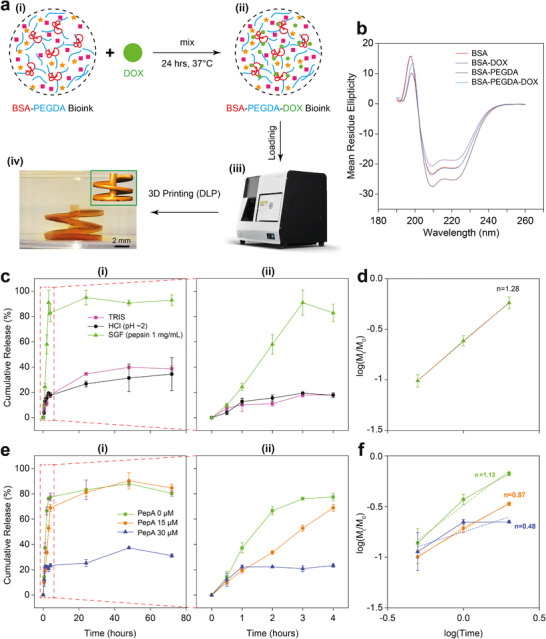
DOX‐BSA binding, 3D‐printed BSA‐PEGDA‐DOX hydrogels, and enzyme‐modulated drug release under gastric‐mimicking conditions. (a) (i‐iii) DOX was mixed with the BSA‐PEGDA complex at 37 °C for 24 h to form the BSA‐PEGDA‐DOX complex. This was then combined with LAP and tartrazine to prepare the BSA‐PEGDA‐DOX bioink. (iv) The successful 3D printing of well‐defined spiral structure demonstrated the high printability of the bioink. (b) CD spectra showing secondary structure changes of BSA upon DOX binding. All samples–including BSA, BSA‐PEGDA, BSA‐DOX, and BSA‐PEGDA‐DOX–exhibited characteristic α‐helix peaks at ∼208 and ∼220 nm. After DOX binding, peak intensities decreased without significant wavelength shifts, indicating reduced α‐helical content. (c,d) DOX release from cylindrical BSA‐PEGDA‐DOX (C*_DOX‐gel_* ∼0.4 mg/mL) hydrogels in different environments TRIS, HCl (pH ∼2), and SGF (pepsin 1 mg/mL, pH ∼2). Pepsin significantly enhanced DOX release, reaching nearly 100% within 4 h, suggesting enzyme‐mediated degradation accelerates drug release. In HCl (pH ∼2) and TRIS, release was slower, with less than 20% released in 4 h and about 40% at 72 h, demonstrating that BSA‐PEGDA hydrogel network stabilizes DOX, reduces burst release, and prolongs drug release. Korsmeyer‐Peppas (SGF) model fitting (*n* = 1.28) indicated a release mechanism controlled by both swelling and enzymatic degradation. (e,f) Cylindrical BSA‐PEGDA‐DOX (C*_DOX‐gel_* ∼ 0.8 mg/mL) hydrogels were incubated at 37°C in 3 mL SGF (pepsin 1 mg/mL, pH ∼2) with C*_PepA_∼*0, 15, and 30 µM, with DOX release quantified by UV‐Vis spectrophotometry at 480 nm over 72 h. Initial release (30 min) was similar across groups due to HCl (pH ∼2)‐induced swelling. Over time, release plateaued at 4 h (C*_PepA_∼*0 µM) and after 24 h (C*_PepA_∼*15 µM) but reached only ∼40% at 72 h (C*_PepA_∼*30 µM). Korsmeyer‐Peppas fitting resulted in *n* = 1.13 (C*_PepA_∼*0 µM), 0.87 (C*_PepA_∼*15 µM), and 0.48 (C*_PepA_∼*30 µM), shifting from enzyme‐driven to diffusion/swelling‐dominated release, highlighting pepsin's regulatory role. All measurements were performed with *n* = 3 independent samples, and data are presented as mean ± SD.

To extend the functionality of BSA‐PEGDA hydrogels beyond morphological responsiveness toward therapeutic applications, we incorporated the widely used anticancer drug DOX, which binds to BSA mainly through hydrophobic interactions, electrostatic attraction, and hydrogen bonding to form a stable BSA‐PEGDA‐DOX complex [78, 79] (Figure 6a‐i–iii). The excellent printability of the BSA‐PEGDA‐DOX bioink was further demonstrated by successfully 3D printing spiral‐shaped structure (Figure 6a‐iv). Notably, the DOX absorption at 480 nm provides a critical basis for simple detection and quantification.

To prove the binding affinity between BSA‐PEGDA and DOX, we introduced gradient‐diluted DOX solutions (0.0075–0.06 mg/mL) while maintaining a constant BSA‐PEGDA solution concentration and analyzed the binding efficiency using fluorescence spectroscopy. This approach leverages the intrinsic fluorescence emission of BSA's tryptophan residues (Trp) at 347 nm, which is quenched upon DOX binding to BSA's hydrophobic pockets, altering the tryptophan microenvironment and shielding its fluorescence center [80, 81]. The results showed that DOX‐induced fluorescence quenching of BSA (0.665 mg/mL) intensified with increasing concentration (see Figure S5a), indicating a strong interaction consistent with a static quenching mechanism. Furthermore, the Stern‐Volmer plot *F*_0_/(*F*_0_ − *F*)*vs*.1/[*drug*] showed excellent linearity (R^2^ = 0.998), from which a high binding constant (K = 1.06 × 10^4^ M^−^^1^) was obtained (see Figure S5b), confirming the formation of a stable BSA‐PEGDA‐DOX complex, consistent with previous studies [78].

During the 3D printing of BSA‐PEGDA‐DOX hydrogels, maintaining consistent BSA‐PEGDA precursor cross‐linking efficiency and concentration was essential. We first prepared a BSA‐PEGDA (2.5–125 mM) bioconjugate mixture, which was then mixed with DOX powder at various concentrations (C*_DOX‐Pre_* ∼0.25, 0.5, 1 mg/mL) at 37°C for 24 h to form BSA‐PEGDA‐DOX complex solutions. This was subsequently combined with LAP and tartrazine, ultimately, 3D printing produced BSA‐PEGDA (2–100 mM)‐DOX hydrogels loaded with various DOX concentrations (C*_DOX‐gel_* ∼0.2, 0.4, and 0.8 mg/mL). After 3D printing and washing, the hydrogels were fully degraded to determine the actual DOX content (M_0_) retained within the matrix.

To investigate the effect of drug binding on the secondary structure of BSA, circular dichroism (CD) spectroscopy was performed on pure BSA, BSA‐PEGDA, BSA‐DOX, and BSA‐PEGDA‐DOX solutions. All samples exhibited two negative peaks at approximately 208 and 220 nm, indicating characteristics of α‐helical structures. After DOX binding, peak intensities slightly decreased but peak positions did not significantly shift (Figure 6b), suggesting a reduction in α‐helical content, consistent with previous reports [82].

To further assess the impact of DOX loading on mechanical properties, we conducted compression tests on BSA‐PEGDA‐DOX hydrogels loaded with various DOX concentrations. We found that the initial stiffness remained stable at 110 kPa across all samples, indicating that DOX loading does not affect the mechanical properties of the hydrogel (see Figure S5d).

Subsequently, we investigated the drug release behavior of DOX‐loaded hydrogels under different conditions. Cylindrical BSA‐PEGDA‐DOX (C*_DOX‐gel_* ∼0.2, 0.4, and 0.8 mg/mL) and PEGDA‐DOX (C*_DOX‐gel_* ∼0.8 mg/mL) hydrogels were prepared via 3D printing and incubated at 37°C in 3 mL of TRIS, HCl (pH ∼2), or SGF (pepsin 1 mg/mL, pH ∼2) for 72 h, with DOX release behavior continuously monitored and recorded. The findings showed that BSA‐PEGDA‐DOX hydrogels exhibited significant release only in SGF, with less than 20% release in the first 30 min, followed by a further increase driven by ongoing enzymatic degradation, reaching nearly 100% release within 4 h and complete degradation of the matrix by 72 h. In contrast, in TRIS and HCl (pH ∼2), the release was below 20% in the first 4 h and approximately 40% after 72 h. However, under HCl (pH ∼2), BSA carries a net positive charge, which typically reduces its binding affinity to DOX (positive charge, pH ∼2)—this is one of the key principles that underlie the pH‐responsive DOX release system [83, 84]. In contrast, in our system, DOX exhibited resistance to acid‐induced burst release, likely due to the formation of strong non‐covalent interactions with BSA‐PEGDA in the precursor solution, along with physical entrapment within the cross‐linked hydrogel network. These interactions collectively limit DOX diffusion under low‐pH conditions, enabling controlled release triggered solely by enzymatic activity in an environment containing both acid and enzyme (Figure 6c; see Figure S5a,e,f, and Video S4). The Korsmeyer‐Peppas model was then applied to analyze the release kinetics of the initial phase, up to 60% cumulative DOX release, revealing that in SGF, the cylindrical hydrogel exhibited a release exponent (*n*) close to or exceeding 0.89, indicative of a super case‐II transport mechanism involving the combined effects of enzymatic degradation and hydrogel swelling [85] (Figure 6d; see Figure S5g,h).

In addition, we tested PEGDA‐DOX (C*_DOX‐gel_* ∼0.8 mg/mL) hydrogels in 3 mL of TRIS, HCl (pH ∼2), and SGF (pepsin 1 mg/mL, pH ∼2). We then compared the color intensity between PEGDA‐DOX and BSA‐PEGDA‐DOX (C*_DOX‐_*_gel_∼0.8 mg/mL) hydrogels. The PEGDA‐DOX samples appeared noticeably lighter in color, indicating that BSA plays a dominant role in DOX retention and loading within the hydrogel matrix (see Figures S5i and S6a). Additionally, PEGDA‐DOX exhibited consistent release rates across all three environments in the first 4 h, dominated by diffusion; subsequently, release in TRIS surpassed that in acidic environments, possibly due to enhanced hydrogen bonding or van der Waals interactions between protonated DOX and the PEGDA network under acidic conditions, reducing release efficiency (see Figure S6a).

To further investigate the regulatory role of pepsin activity in DOX release, we immersed cylindrical BSA‐PEGDA‐DOX (C*_DOX‐gel_* ∼0.8 mg/mL) hydrogels in 3 mL of a mixed solution with SGF (pepsin 1 mg/mL, pH ∼2) and varying PepA concentrations (C*_PepA_∼*0, 15, and 30 µM), incubated at 37°C for 72 h, and measured DOX release at different time points. It was observed that in the first 30 min, all three groups exhibited release below 20%, primarily driven by hydrogel swelling induced by HCl (pH ∼2); over time, DOX release significantly lagged with increasing PepA concentration, with the C*_PepA_∼*0, 15 µM groups reaching a plateau and nearly 100% release after 4 and 24 h, respectively, while the C*_PepA_∼*30 µM group achieved only ∼40% total release after 72 h, consistent with HCl (pH ∼2) group, indicating complete inhibition of pepsin activity by PepA (Figure 6e; see Figure S6b). Fitting the data with the Korsmeyer‐Peppas equation revealed n values of 1.13 and 0.87 for the C*_PepA_∼*0, 15 µM groups, respectively, indicating enzyme degradation as the dominant mechanism, while the C*_PepA_∼*30 µM group had an n value of ∼0.48, driven by diffusion and swelling (Figure 6f). These findings confirm the critical role of pepsin activity in regulating DOX release.

### 2.7 Enzyme‐Responsive DOX Release Coupled with Autonomous Shape Morphing in 3D‐Printed BSA‐PEGDA Hydrogel Actuators

**FIGURE 7:**
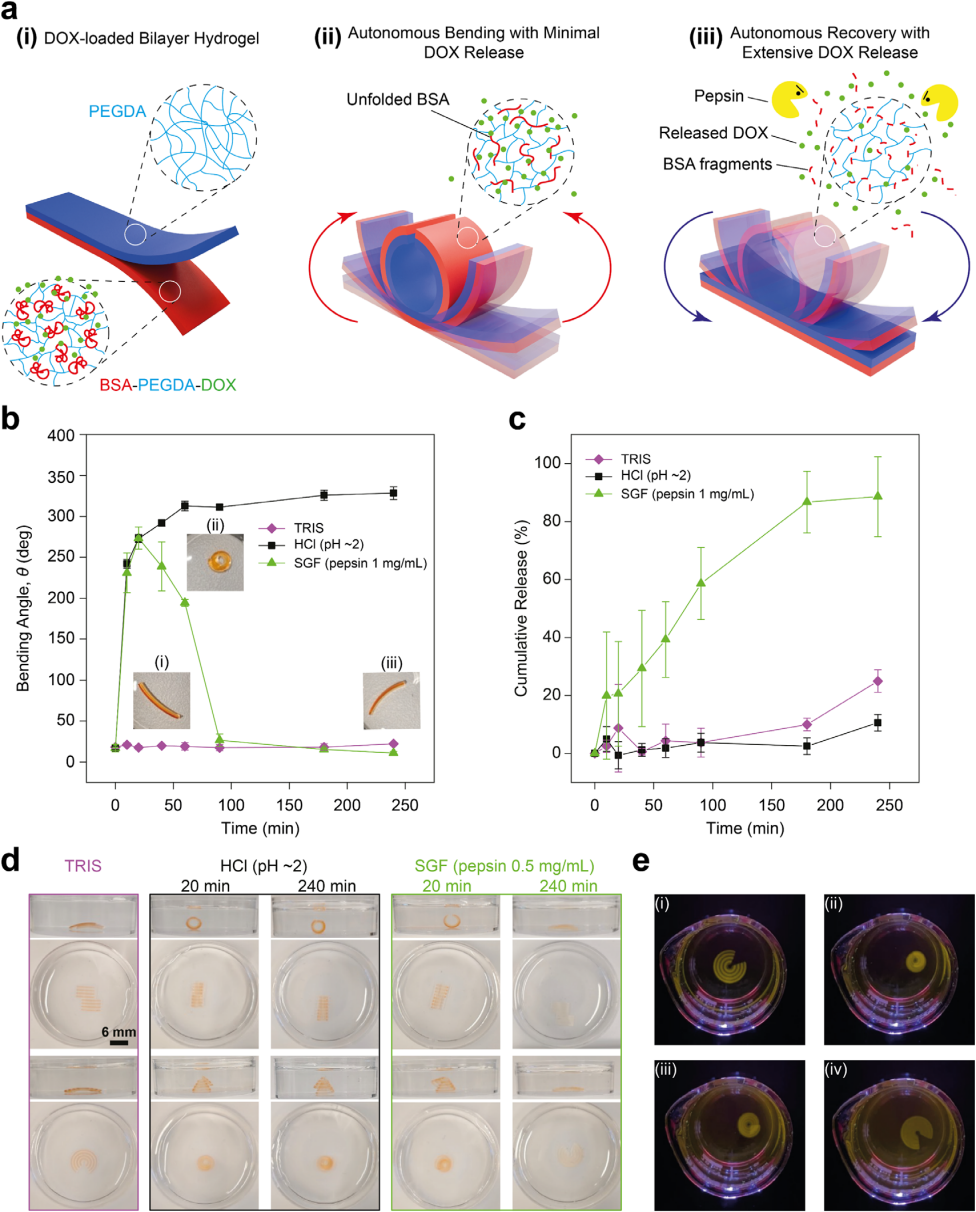
Autonomous, enzyme‐regulated shape morphing and drug release from 3D‐printed BSA‐PEGDA‐DOX bilayer hydrogel actuators. (a) Schematic illustration of the autonomous shape‐morphing and drug release behavior of the BSA‐PEGDA‐DOX bilayer hydrogel actuator. (i) Initially, the hydrogel consists of a PEGDA passive layer and a DOX‐loaded BSA‐PEGDA active layer. (ii) In acidic gastric fluid (pH ∼2), BSA unfolds, inducing autonomous bending, with minimal DOX release. (iii) As pepsin‐mediated degradation increases, BSA undergoes enzymatic degradation, triggering shape recovery and extensive DOX release. (b) Shape morphing and DOX release of BSA‐PEGDA‐DOX (C*_DOX‐gel_* ∼0.8 mg/mL) bilayer hydrogels in different environments. The hydrogels swelled and bent rapidly within 20 min in HCl (pH ∼2). In SGF (pepsin 1 mg/mL, pH ∼2), they initially bent and subsequently returned to their original shape after 60 min. In contrast, no significant shape change occurred in TRIS. (c) The SGF (pepsin 1 mg/mL, pH ∼2) condition significantly accelerated hydrogel degradation and DOX release, while TRIS and HCl (pH ∼2) conditions exhibited slower release. (d) DOX release from complex‐shaped BSA‐PEGDA‐DOX (C*_DOX‐gel_* ∼0.8 mg/mL) hydrogel actuators in different environments. In TRIS, the hydrogel remained stable (orange color). In acidic conditions, it transformed from flat to 3D structure but did not release DOX (orange color). Under SGF (pepsin 0.5 mg/mL, pH ∼2) treatment, it transitioned from flat to 3D structure and then back to flat while fully releasing DOX (transparent). (e) The pyramid actuator first underwent acid‐triggered bending, followed by relaxation as pepsin gradually degraded more BSA in SGF (pepsin 0.5 mg/mL, pH ∼2). Images were recorded under 405 nm violet‐light illumination to monitor DOX fluorescence. The diminishing fluorescence signal reflects enzyme‐triggered DOX release from the BSA‐PEGDA network (see Video S5). All measurements were performed with *n* = 3 independent samples, and data are presented as mean ± SD.

Building on the established DOX release behavior, we further explored the integration of drug release with autonomous shape morphing, aiming to develop a multifunctional bilayer actuator capable of autonomous reversible shape morphing and enzyme‐responsive DOX release in SGF (Figure 7a). We prepared BSA‐PEGDA‐DOX (C*_DOX‐gel_* ∼0.8 mg/mL)/PEGDA bilayer hydrogels via 3D printing and tested them in of TRIS, HCl (pH ∼2), and SGF (pepsin 1 mg/mL, pH ∼2). Our findings demonstrated that the system achieved autonomous shape morphing (Figure 7b; see Figure S6c), exhibiting bending behavior consistent with that of the BSA‐PEGDA/PEGDA bilayer actuator, and pepsin‐responsive DOX release in SGF, with nearly 100% release within 4 h, while showing minimal release in TRIS and HCl (pH ∼2) environments (Figure 7c). To explore the synergistic effects of different shapes on shape morphing and DOX release, we tested bamboo‐shaped and pyramid‐shaped BSA‐PEGDA‐DOX (C*_DOX‐gel_* ∼0.8 mg/mL)/PEGDA hydrogel actuators. The evidence indicated that under SGF (pepsin 0.5 mg/mL, pH ∼2) action, these hydrogels achieved autonomous reversible shape morphing, with the BSA‐PEGDA‐DOX layer transitioning from light red to transparent, indicating DOX release; in contrast, in HCl (pH ∼2), the hydrogels only formed pyramid‐like or curled shapes without recovery, exhibiting minimal DOX release and negligible color change (Figure 7d,e; see Video S5). This phenomenon suggests that drug release and autonomous shape morphing can operate independently without interference, successfully yielding a hydrogel actuator with both enzyme‐responsive drug release and autonomous reversible shape morphing capabilities. This actuator enables precise enzyme‐responsive DOX release in SGF and expands its potential for biomimetic medical applications through shape morphing, offering new insights for precise drug delivery and intelligent system design.

## 3 Discussion

Hydrogels have achieved significant advances in shape morphing, drug release, and mucosal adhesion, demonstrating great potential for gastric drug retention and prolonged therapeutic efficacy. However, challenges remain regarding actuator fabrication complexity, production cost, and the limited responsiveness of hydrogels under physiological conditions. In the stable in vivo environment —characterized by constant temperature, hydration, and pH—most hydrogels undergo only a single activation after implantation, resulting in irreversible deformation [86]. Furthermore, many systems rely on external stimuli and manual intervention to achieve sequential morphing, which is technically challenging in the gastric milieu. Although photosensitive hydrogels enable reversible control, their in vivo application is restricted by the insufficient light penetration depth through biological tissues [86]. In contrast, this study simplifies the fabrication process via 3D printing and utilizes the dual endogenous acid‐enzyme signals of gastric fluid to achieve autonomous and reversible shape morphing and mucosal adhesion without external intervention, significantly expanding the applicability and functional boundary of the actuator under complex physiological conditions.

**FIGURE 8:**
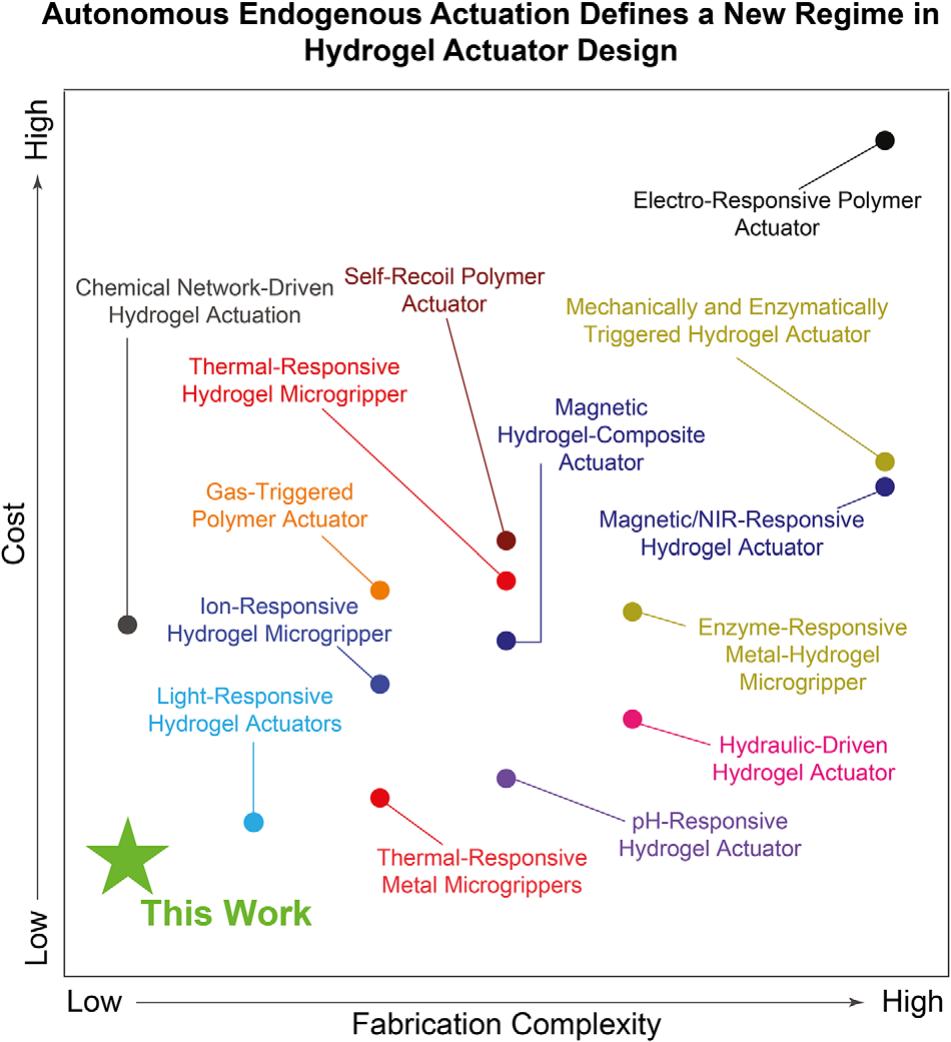
Ashby plot comparing fabrication complexity and material cost across representative hydrogel and polymer actuator systems. “Cost” is defined as the total consumable material expenditure required to fabricate one actuator, excluding instrument usage and labor. “Fabrication complexity” is quantified as the number of discrete processing steps needed to produce a functional device, with each independent step counted once. Our endogenous biochemical‐logic hydrogel actuator (green star) occupies the uniquely advantageous “low‐complexity, low‐cost” regime, distinguishing it from existing systems that rely on external triggers (including thermal [75, 87], hydraulic [88], light [7], electrical [89], pH [90], magnetic [17, 91], self‐recoiling [39], gas‐driven [92], ionic [93], enzymatic [47, 52] or chemically engineered environments [94]) or multi‐step microfabrication. This position highlights the innovation of a fully autonomous, physiology‐driven actuation mechanism that simplifies fabrication while enabling programmable behaviors within a clinically relevant environment.

Here, we propose a generalizable design framework for autonomous multi‐stage actuators, in which the deformation sequence can be programmably tuned by controlling the environmental response rates of protein‐polymer component. The core structure consists of a BSA‐PEGDA bioconjugate network that integrates the biocompatibility and environmental responsiveness of natural proteins with the structural tunability and mechanical robustness of synthetic polymers. A printable BSA‐PEGDA bioink was developed to fabricate bilayer actuator structures with predefined deformation pathways, enabling high‐resolution and low‐cost manufacturing (Figure 8).

The structural responsiveness of BSA imparts the hydrogel with adaptive regulation toward the acid‐enzyme environment of gastric fluid, exhibiting a two‐stage response of acid‐induced rapid swelling and enzyme‐mediated gradual softening in SGF (Figure 2a,b). Cryo‐SEM revealed enlarged pore size and partial network fracture (Figure 2e), confirming its dynamic microstructural adaptability. This synergistic mechanism enables the actuator to autonomously execute a range of biomedical functions, including catch‐and‐release motion and reversible adhesion to simulated mucus layers, along predefined deformation pathways under physiological gastric conditions without external stimuli and manual intervention (Figures 4 and 5), while maintaining excellent biocompatibility (Figure 2f). The actuation mechanism is driven by acid‐induced unfolding of BSA, which causes rapid swelling of the outer BSA‐PEGDA layer, while the inner PEGDA layer remains stable, leading to a swelling mismatch that induces bending. Subsequently, enzymatic degradation gradually reduces the stiffness of the BSA‐PEGDA layer, allowing it to recover its original shape under the elastic recovery of the PEGDA layer (Figure 3a). In addition, the introduction of the pepsin inhibitor, PepA, allows precise regulation of actuation duration. Increasing the concentration of PepA effectively delays BSA degradation, thereby extending the recovery process and actuator lifetime. This biochemical modulation transforms the system from a traditional 4D‐printed structure into a 5D rate‐programmable platform, in which the actuation kinetics are primarily governed by enzyme reaction rates (Figure 3d).

In terms of drug loading, this study employs BSA as a natural, non‐toxic carrier that incorporates small‐molecule drugs non‐covalently through electrostatic, hydrogen bonding, and hydrophobic interactions, forming a BSA‐PEGDA‐drug bioink (Figure 6a; Figure S5). Compared with traditional drug‐loading methods, such as xerogel loading, immersion, or surface adsorption [95], this system avoids structural collapse [96], burst release, and heterogeneity [97, 98, 99], and overcomes the limitation of incompatibility with high‐resolution 3D printing. By embedding the drug directly within the protein‐polymer network rather than post‐loading onto the surface, the formulation establishes an intrinsic link between structural evolution and release behavior. Importantly, this in situ drug encapsulation strategy directly couples the mechanical dynamics of the hydrogel with its drug‐release kinetics.

This structural‐pharmacological coupling becomes evident when examining the time‐dependent mechanical response of the hydrogel under gastric conditions. We observed that the stiffness of the BSA‐PEGDA hydrogel sharply decreased within 0.5 h in SGF (pH ∼2) due to acid‐induced unfolding of BSA and reached a minimum after approximately 2 h. From a pharmacokinetic and pharmacodynamic perspective, this temporally programmed response is particularly advantageous for short half‐life drugs such as DOX, which exhibits a therapeutic half‐life of less than 0.5 h during the initial distribution phase, as it is rapidly cleared from plasma and extensively distributed into tissues [100]. Excessively slow release would therefore fail to maintain effective concentrations.

Building upon this correlation between structural relaxation and release, our system achieves precise pharmacokinetic matching through a “transient retention‐enzyme‐gated release” process: In the initial stage, acid‐induced swelling enhances mucosal adhesion while restricting diffusion (release rate <20%), thereby promoting local retention and significantly reducing burst release; and with time, continuous enzymatic hydrolysis leads to further BSA degradation and enlarged pore structures, enabling nearly 100% sustained release within about 4 h and autonomous detachment (Figure 7c). Based on the Korsmeyer‐Peppas model, the release process is mainly governed by enzymatic degradation (Figure 6d) and can be precisely modulated by PepA (Figure 6e,f). This two‐stage kinetic logic translates molecular conformational dynamics into a programmable pharmacological profile.

Unlike previously reported mucosal grippers [87], our system autonomously detaches after drug release, avoiding excessive mucosal retention and local irritation, while enabling time‐programmable regulation through endogenous biochemical signals, thereby extending gastric residence and drug release duration. From a broader perspective of oral drug delivery, existing stimuli‐responsive hydrogels generally suffer from insufficient mechanical strength, limited physiological adaptability, and weak coupling between degradability and biocompatibility [101, 102, 103]; traditional formulations (immediate‐release, enteric‐coated, or controlled‐release) also face challenges such as acid/enzyme‐triggered burst release, short gastric retention, and complex preparation processes [104, 105]. In contrast, the BSA‐PEGDA‐DOX hybrid hydrogel actuator developed in this study integrates the complementary advantages of natural proteins and synthetic polymers, achieving high biocompatibility, facile fabrication, excellent structural uniformity, high drug‐loading capacity, and enzyme‐responsive release, thereby offering a promising strategy for gastric retention and controlled drug delivery.

The scalability and translational potential of DLP 3D printing deserve special attention, as they directly determine the feasibility of clinical manufacturing and future commercialization. Accordingly, we evaluated its potential for clinical‐scale manufacturing, although DLP offers high precision and reproducibility, clinical‐grade production remains constrained by protein stability, formulation homogeneity, printing throughput, and bioink reusability. The structure of BSA is highly sensitive to temperature and UV exposure, requiring precise control of light intensity and exposure time to prevent denaturation. Non‐uniform formulation or inconsistent cross‐linking may lead to variations in deformation, which can be mitigated by optimizing mixing and printing parameters to improve homogeneity. Current benchtop DLP printers have limited throughput, which can be enhanced through multi‐projection modules [106] or continuous photopolymerization printing architectures [107, 108]. Clinical translation and scale‐up requires batch‐to‐batch reproducibility verification under GMP conditions, strict control of residual monomers, sterilization compatibility, and long‐term stability. Since BSA‐PEGDA contains natural protein components, it demands higher certification standards and batch consistency compared to purely synthetic systems. Establishing standardized synthesis and verification protocols is therefore critical. Moreover, bioink reusability and storage stability are key to cost reduction; long‐term storage or repeated use may lead to viscosity changes or protein denaturation, necessitating systematic evaluation of rheology and photopolymerization kinetics.

Focusing on the limitations and future optimization of the system, several key issues remain to be explored. The current actuator exhibits a single autonomous actuation cycle, which is advantageous for gastric applications—where a single catch‐and‐release event is sufficient for stable adhesion, sustained release, and autonomous detachment. However, achieving multiple reversible cycles would greatly expand its potential for multi‐cycle operation. Future designs may introduce reversible covalent bonds [109, 110] or supramolecular interactions [111] to enable repeatable actuation in non‐proteolytic environments. Meanwhile, repeated actuation capabilities would be more relevant for devices that can be reloaded with additional drugs after complete release. In our case, the actuator is designed for single‐use operation, as it cannot be reloaded once the drug release process is completed. The current bending speed is relatively slow, limiting its application in rapid‐response scenarios. To enhance actuation efficiency, future work will focus on constructing dual‐responsive bilayer structures that couple acid‐enzyme cues with additional stimuli (temperature, pH, or enzyme‐sensitive components), using synergistic stress gradients to accelerate deformation and achieve faster, more controllable actuation. These optimization directions aim to transform the current single‐use gastric actuator into a reprogrammable soft‐material platform with autonomous repeatable actuation. Mechanical testing and finite element modeling will be combined to evaluate durability and guide structural optimization.

During multilayer hydrogel fabrication, material infiltration and light scattering can cause unintended cross‐linking within the BSA‐PEGDA layer, reducing its enzymatic degradability by pepsin and hindering complete shape recovery (Figure S7). Possible solutions include protein stabilization, optimized light exposure, and interfacial barriers to prevent interlayer mixing.

At present, the study remains limited to simulated and ex vivo gastric fluid experiments, and its in vivo performance has yet to be verified. Therefore, this work represents a proof‐of‐concept preclinical exploration, demonstrating autonomous staged actuation and controlled drug release under physiologically relevant conditions. In the next stage, in vivo porcine studies will be conducted to evaluate real‐time actuation, mucosal retention, and drug release behavior in the native gastric environment, supported by endoscopic imaging, histology, and pharmacokinetic analyses. This will help elucidate physiological factors such as gastric motility, mucus turnover, enzymatic activity, and immune response, which are essential for clinical translation.

Finally, regarding biosafety, the fragments generated from BSA‐PEGDA hydrogel degradation in SGF are not ideal for direct cytocompatibility evaluation, as the neutralization process conducted in vitro results in relatively high (cell incompatible) NaCl concentrations and may create a mildly hyperosmotic environment that can be detrimental to the cell compatibility results. Previous reports have shown that even moderate increases in osmolarity can significantly reduce cell viability [112]. In this study, HT29‐MTX cells cultured with intact BSA‐PEGDA hydrogels for seven days maintained viability above 80%, confirming the very good intrinsic compatibility of these materials. The degradation products themselves exhibited no detectable chemical cytotoxicity and, under physiological conditions, would be rapidly diluted in the gastrointestinal fluids and cleared by gastric motility and luminal flow. Accordingly, the 7‐day static exposure assay represents a conservative, worst‐case evaluation designed to overestimate (rather than underestimate) potential cytotoxic effects. Collectively, these findings verify the good cytocompatibility of the BSA‐PEGDA system while underscoring the need for further in vivo studies to validate its long‐term biocompatibility.

In summary, this study presents the first hydrogel actuator capable of sequentially responding to two endogenous gastric signals to achieve dual‐stage morphing and enzyme‐gated drug release without external control. This strategy bridges the gap between stimuli responsiveness and physiological adaptability, providing a general design framework for next‐generation autonomous soft actuators capable of stable operation under complex biological environments.

### 3.1 Statistical Analysis

All quantitative measurements were performed using *n* = 3 independent samples, and results are reported as mean ± standard deviation (SD). No additional data preprocessing, normalization, or statistical hypothesis testing was applied. Sample size (n) and data presentation format are also provided in each corresponding figure caption. Statistical analysis and plotting were performed in OriginLab software.

## Author Contributions

**Luai R. Khoury** led the project and conceived the idea. **Luai R. Khoury** and **Yuchen Liu** designed the experiments. **Yuchen Liu** performed the experiments, conducted data analysis, and prepared and wrote the initial manuscript draft. **Harischandra Potthuri** performed the cell cytocompatibility experiment. **Yuchen Liu**, **Alejandro Sosnik**, and **Luai R. Khoury** revised the manuscript for intellectual content. All authors read and approved the final version of the manuscript.

## Conflicts of Interest

The authors declare no conflict of interest.

## Supporting information




**Supporting File 1**: adma72176‐Sup‐0001‐SuppMat.docx.

**Supporting File 2**: adma72176‐Sup‐0002‐VideoS1.mp4.

**Supporting File 3**: adma72176‐Sup‐0003‐VideoS2.mp4.

**Supporting File 4**: adma72176‐Sup‐0004‐VideoS3.mp4.

**Supporting File 5**: adma72176‐Sup‐0005‐VideoS4.mp4.

**Supporting File 6**: adma72176‐Sup‐0006‐VideoS5.mp4.

## Data Availability

The data that support the findings of this study are available from the corresponding author upon reasonable request.;
