# Supplementary material for: Autonomous Hydrogel Actuators Programmed by Endogenous Biochemical Logic for Dual‐Stage Morphing and Drug Release
Source: Adv Mater. 2026 Jan 21;38(12):e16809. doi: 10.1002/adma.202516809 (PMC12933017; doi:10.1002/adma.202516809)
Supplement: Supplementary file 1 — Supporting File 1: adma72176‐Sup‐0001‐SuppMat.docx. [file ADMA-38-e16809-s005.docx]

**Autonomous Hydrogel Actuators Programmed by Endogenous Biochemical Logic for Dual-Stage Morphing and Drug Release**

Yuchen Liu, Harischandra Potthuri, Alejandro Sosnik, Luai R. Khoury

Department of Materials Science and Engineering, Technion – Israel Institute of Technology, Haifa, Israel

Supplementary Information

# ****Results****


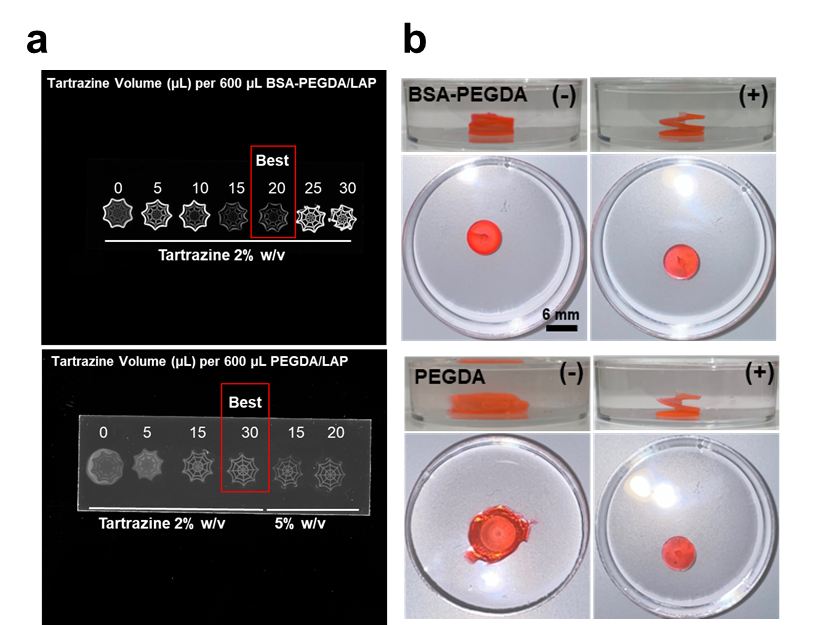


**Figure S1. Optimization of Tartrazine Concentration for High-Fidelity 3D Printing of BSA-PEGDA and PEGDA Hydrogels.** (a) Tartrazine concentrations were optimized using 3D-printed spider-web test structures. Tartrazine stock solutions (2% and 5% w/v in TRIS) were added to 600 µL precursor mixtures at defined volumes: 0~30 µL of the 2% stock for BSA-PEGDA/LAP, and 0~30 µL of the 2% stock plus 15 and 20 µL of the 5% stock for PEGDA/LAP. Printed BSA-PEGDA constructs were immersed in TRIS containing ANS (30 µM) for 10 min and imaged to evaluate network fidelity. Optimal formulations were identified as 30:1 (BSA-PEGDA/LAP : Tartrazine 2% w/v) and 20:1 (PEGDA/LAP : Tartrazine 2% w/v). (b) Spiral structures printed using these optimized ratios exhibited markedly improved geometric accuracy when tartrazine was incorporated (+), compared to bioinks lacking tartrazine (–), demonstrating the essential role of tartrazine in enhancing print precision.


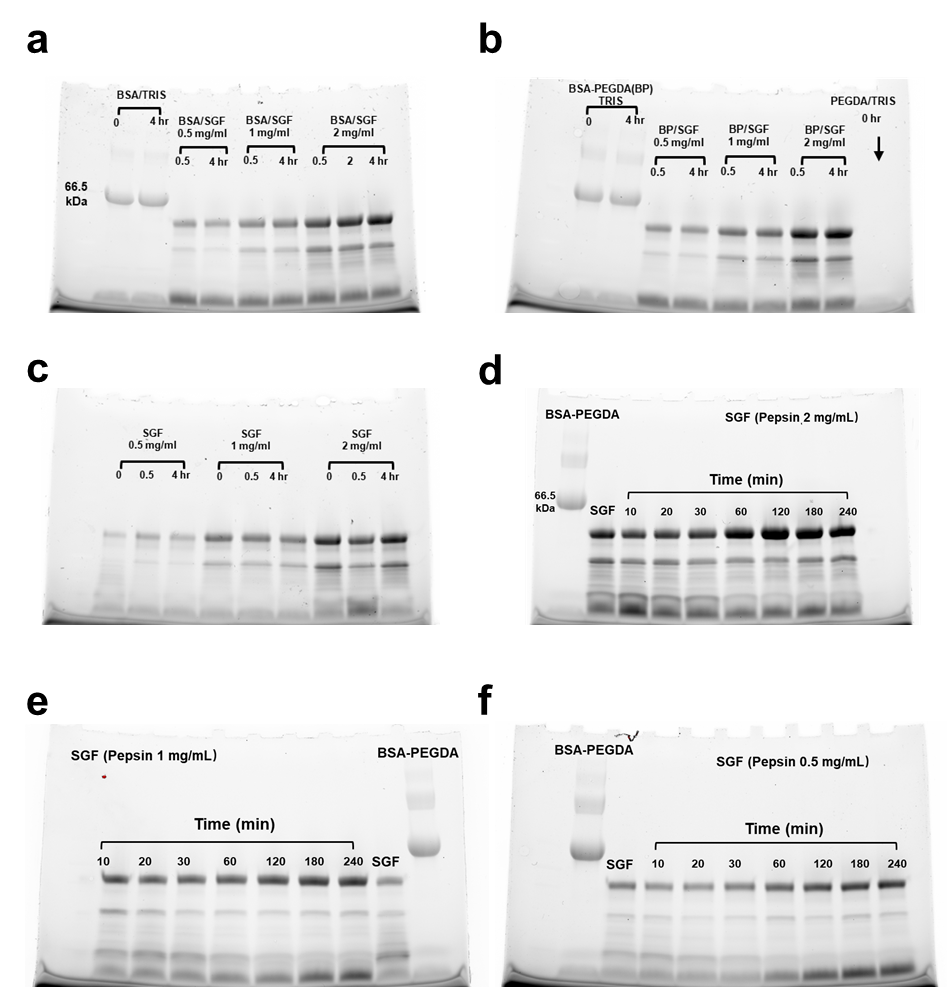


**Figure S2. SDS-PAGE analysis of BSA degradation and release from hydrogels in simulated gastric fluid (SGF) at 37 °C.** (a-c) Comparison of pure BSA solutions (2 mM) and BSA-PEGDA precursor solutions (2-100 mM) exposed to identical SGF conditions (pepsin 0.5 1, and 2 mg/mL, pH ~2, 37 °C) versus TRIS controls. Both pure BSA and BSA-PEGDA solutions were fully degraded within 4 h, and their fragmentation profiles closely matched those observed for the corresponding hydrogel samples. PEGDA-only (100 mM) hydrogels incubated in TRIS exhibited no detectable protein bands, and SGF blanks (without BSA) showed only faint background bands, confirming that the intensified SDS-PAGE signals originate exclusively from pepsin-mediated cleavage of BSA. (d-f) Full-lane SDS-PAGE analysis of BSA degradation and release from BSA-PEGDA hydrogels (2-100 mM) incubated in SGFs (pepsin 0.5, 1, 2 mg/mL, pH ~2) at 37 °C for up to 4 h. Adjacent lanes include the corresponding BSA-PEGDA precursor solutions and SGF samples for direct comparison of degradation patterns.


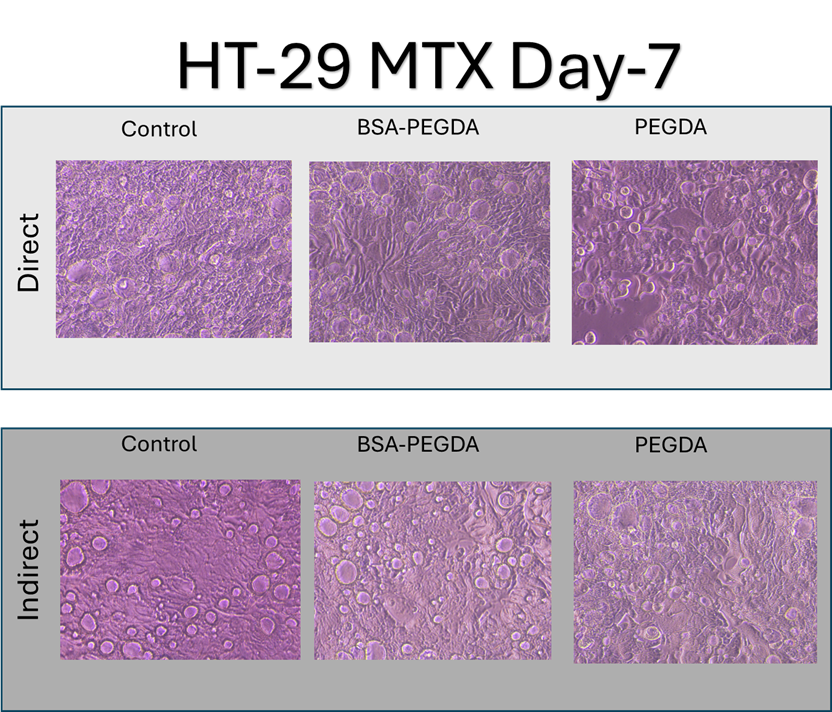


**Figure S3. Cytocompatibility assessment of BSA-PEGDA and PEGDA hydrogels.** Bright-field micrographs of HT29-MTX cells after 7 days of culture in the presence of BSA-PEGDA (2-100 mM) and PEGDA (200 mM) hydrogels under **direct-contact** (top) and **indirect-contact** (bottom) conditions. In all cases, cells exhibited normal morphology and confluence comparable to untreated controls, indicating that both hydrogel types are highly cytocompatible with the mucus-secreting intestinal epithelial cell line HT29-MTX.


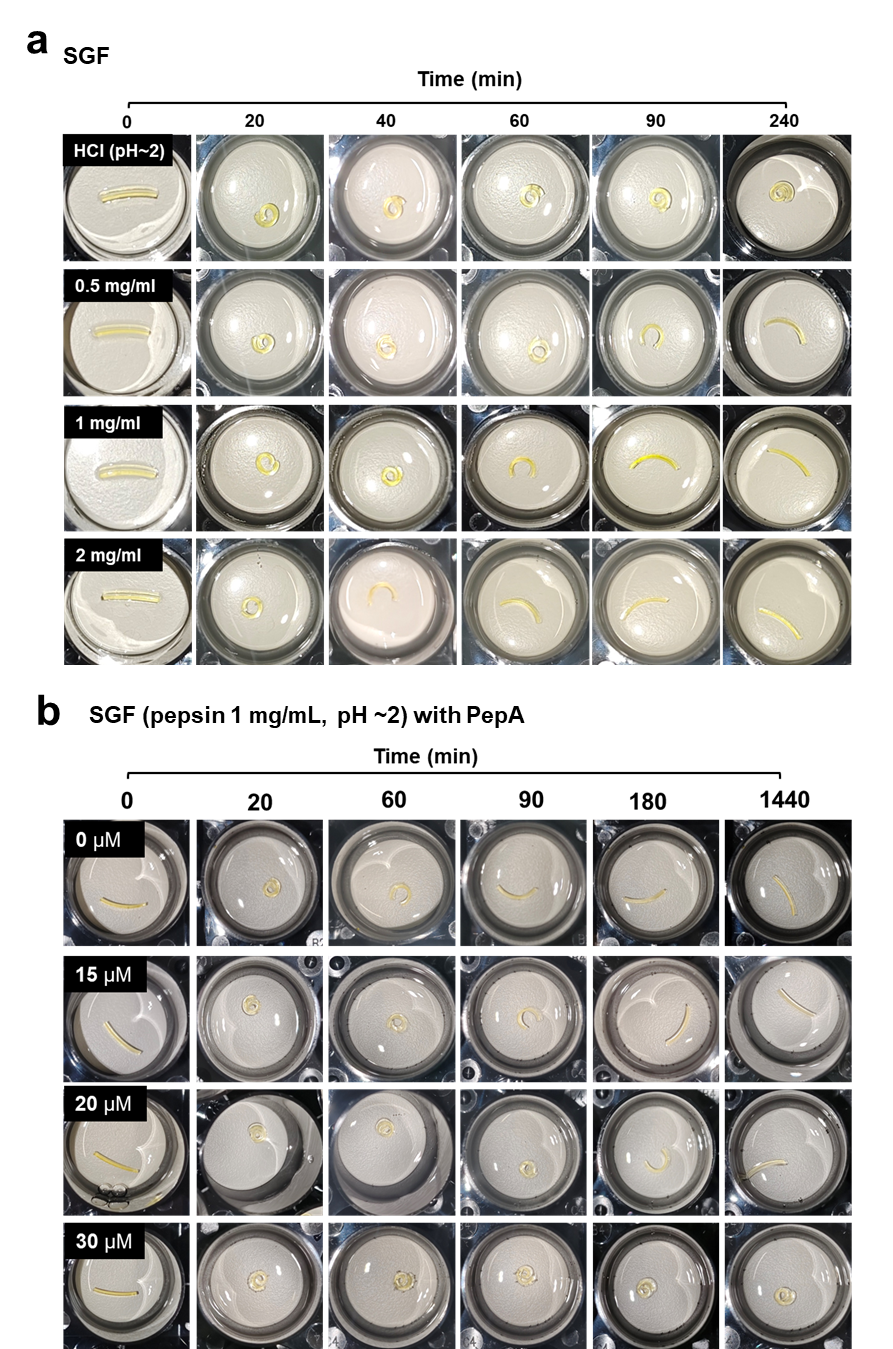


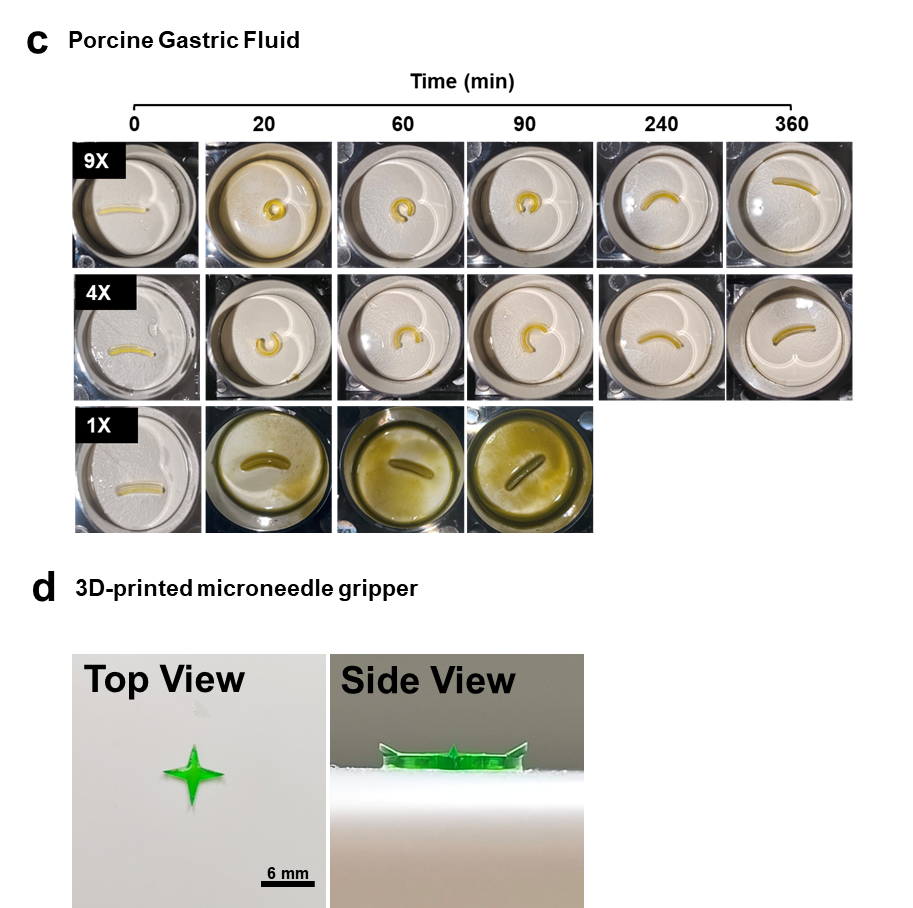


**Figure S4. Shape‐morphing behavior of BSA-PEGDA hydrogel actuators under gastric‐mimicking conditions at 37 °C.** (a) Time-dependent shape transformations of the actuators in SGFs (pepsin 0.5, 1, 2 mg/mL, pH ~2) and in HCl (pH ~2). (b) Shape morphing in SGF (pepsin 1 mg/mL, pH ~2) as a function of PepA concentration (0, 15, 20, and 30 µM). (c) Shape-morphing profiles in porcine gastric fluid diluted to 1×, 4×, and 9×. (d) Stereomicroscopic image of a 3D-printed microneedle-integrated gripper (diameter 6 mm; total height 1.5 mm; microneedle height 0.9 mm), illustrating the geometric versatility achievable with the BSA-PEGDA platform.


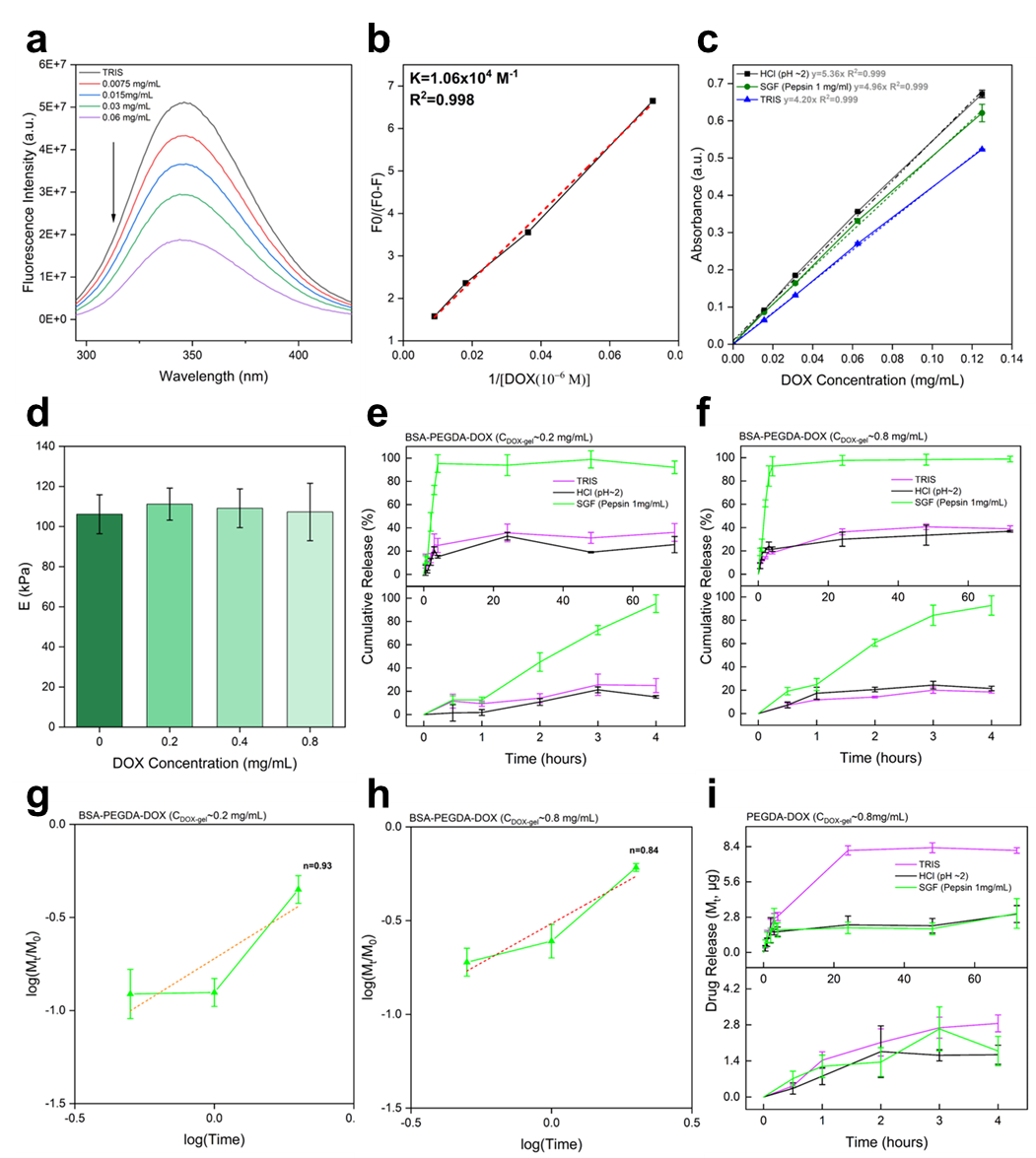


**Figure S5. Analysis of DOX release from BSA-PEGDA-DOX hydrogels under various environmental conditions.** (a,b) Fluorescence spectroscopy of BSA-PEGDA-DOX interactions. At fixed BSA-PEGDA concentration, increasing DOX (0.0075~0.06 mg/mL) quenched BSA fluorescence at 347 nm, reflecting DOX binding within the hydrophobic pocket and altering the tryptophan microenvironment. The decrease in intensity followed a static-quenching trend. A linear plot of *F₀/(F₀–F)* vs. 1/[DOX] (R² = 0.998) yielded a binding constant of *K* = 1.06 × 10⁴ M⁻¹, confirming formation of a stable BSA-PEGDA-DOX complex. (c) Calibration curves of DOX in HCl (pH ~2), SGF (pepsin 1 mg/mL, pH ~2), and TRIS. (d) Young’s modulus of BSA-PEGDA-DOX (C*_DOX-gel_*~0.2, 0.4, 0.8 mg/mL) hydrogels. No significant change in stiffness was observed compared to BSA-PEGDA controls, with *E*~110 kPa across all formulations. (e,f) Cumulative DOX release from BSA-PEGDA-DOX hydrogels (C*_DOX-gel_*~0.2 and 0.8 mg/mL) incubated at 37 °C in HCl (pH ~2), TRIS, and SGF (pepsin 1 mg/mL, pH ~2). Substantial release occurred only in pepsin-containing media, reaching nearly 100% within 4 h. (g,h) Peppas–Korsmeyer fitting of DOX release profiles in SGF. The release exponent (*n*) approached or exceeded 0.89, indicating transport governed by combined swelling and enzymatic degradation. (i) Cumulative DOX release (M_t_) from PEGDA-DOX hydrogels (C*_DOX-gel_*~0.8 mg/mL). Over extended incubation, TRIS produced the highest total release, whereas acidic conditions modulated DOX-hydrogel interactions and slowed long-term release. During the first 4 h, release behavior across conditions remained similar.


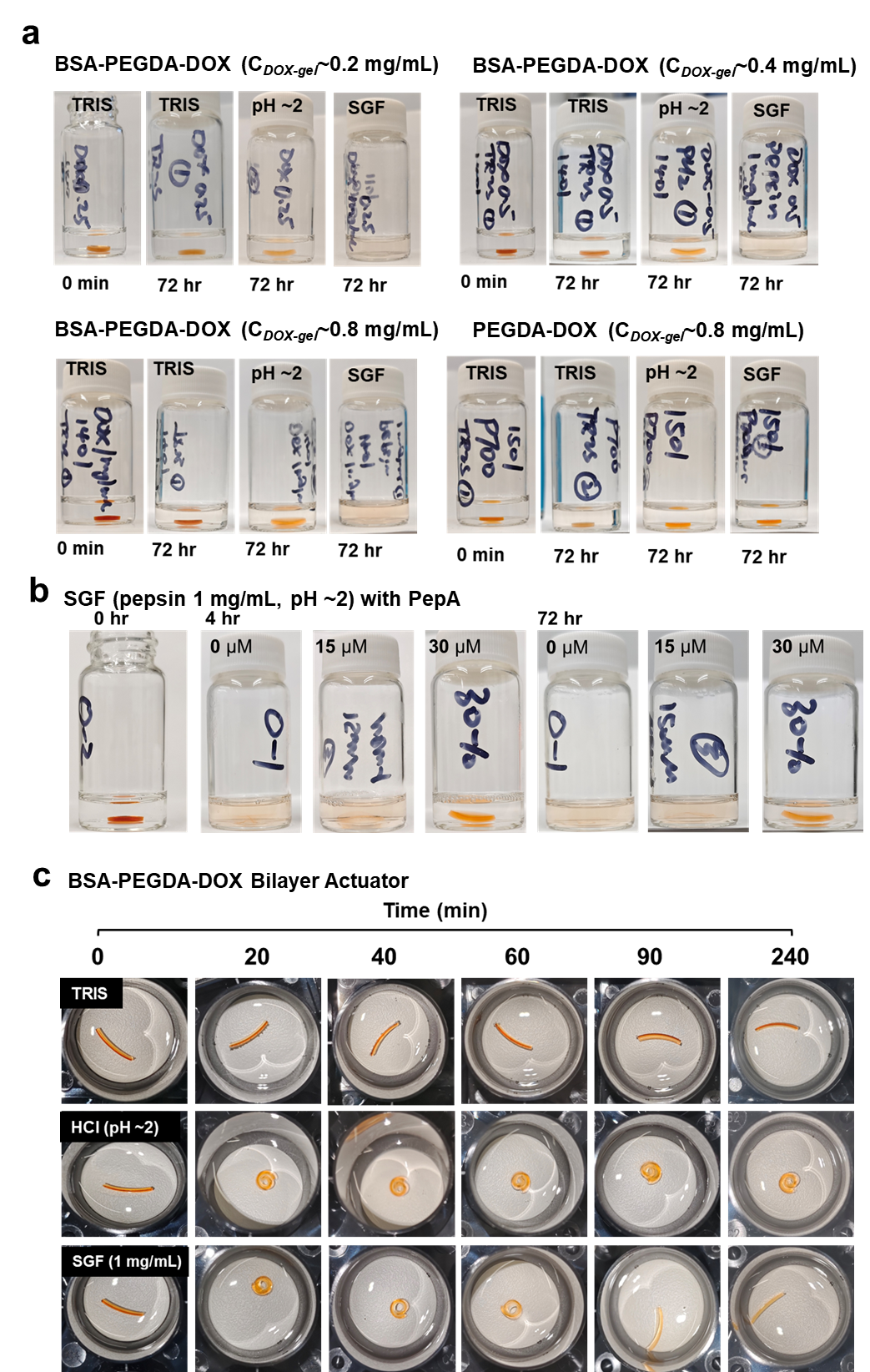


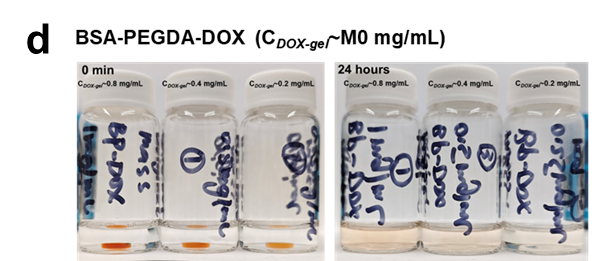


**Figure S6. Degradation, dissolution, and shape-morphing behavior of BSA-PEGDA-DOX hydrogels and corresponding actuators at 37 °C.** (a) Degradation and dissolution behavior of BSA-PEGDA-DOX and PEGDA-DOX hydrogels after 72 h of incubation in TRIS, HCl (pH ~2), and SGF (pepsin 1 mg/mL, pH ~2). BSA-PEGDA-DOX hydrogels displayed minimal changes in TRIS, swelling and color fading in HCl, and near-complete degradation in SGF. PEGDA-DOX hydrogels exhibited gradual color fading in TRIS and only minor visual changes under acidic conditions. (b) Degradation profiles of BSA-PEGDA-DOX (C*_DOX–gel_*~0.8 mg/mL) hydrogels after 4 h and 72 h in SGF (pepsin 1 mg/mL, pH ~2) containing varying PepA concentrations (0, 15, 30 µM). After 72 h, samples at 0 and 15 µM PepA were almost fully degraded, whereas hydrogels at 30 µM PepA retained structural integrity, similar to samples exposed only to acidic conditions. These results demonstrate that PepA strongly inhibits pepsin activity, thereby delaying DOX release and preventing hydrogel breakdown. (c) Shape-morphing behavior of BSA-PEGDA-DOX actuators (C*_DOX-gel_*~0.8 mg/mL) in TRIS, HCl (pH ~2), and SGF (pepsin 1 mg/mL, pH ~2). Actuators remained stable in TRIS but underwent pronounced shape transformations in HCl and SGF. (d) Complete degradation of BSA-PEGDA-DOX hydrogels (C*_DOX–gel_*~0.2, 0.4, 0.8 mg/mL) in SGF (pepsin 1 mg/mL, pH ~2) after 24 h at 37 °C. The resulting solutions were collected for quantification of the initial DOX loading (M₀).


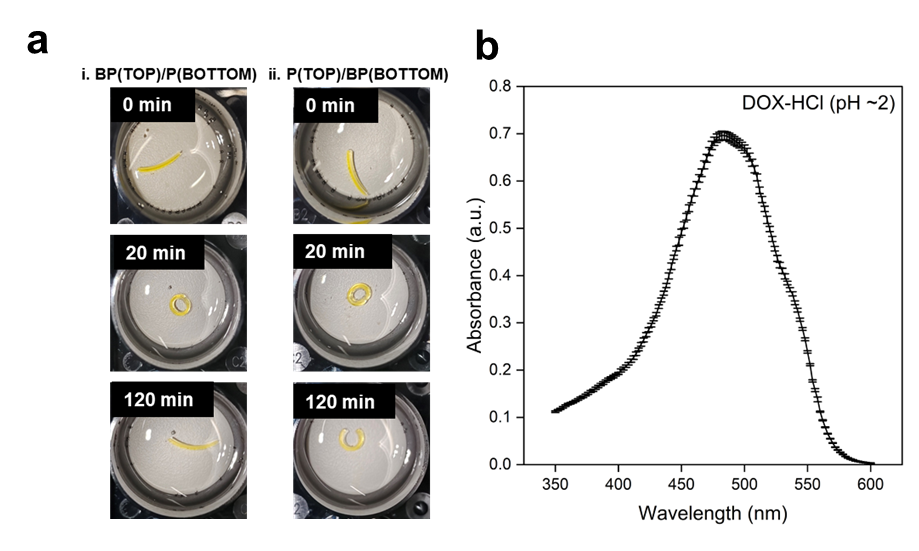


**Figure S7. Influence of material printing sequence on actuator shape-morphing behavior and absorbance spectrum of DOX in acidic conditions.** (a) Proper sequencing in bilayer 3D printing is essential for reliable actuator performance. The PEGDA (200 mM) layer must be printed first, followed by the BSA-PEGDA (2-100 mM) layer. Reversing the order causes the uncrosslinked PEGDA solution to infiltrate the underlying BSA-PEGDA layer, disrupting its network formation. In addition, altered light refraction during curing can induce excessive crosslinking in the BSA-PEGDA region, increasing stiffness and impairing its degradation-driven recovery. Experiments in SGF (pepsin 2 mg/mL, pH ~2) show that printing sequence strongly affects the resulting shape-morphing behavior. (b) Absorbance spectrum of DOX (0.125 mg/mL) in HCl (pH ~2), confirming that its characteristic absorption peak at 480 nm remains unchanged under acidic conditions.

**Video S1:** Shape morphing behavior of BSA-PEGDA bilayer hydrogel actuators in SGF (pepsin 0.5 mg/mL, pH ~2) and HCl (pH ~2) at 37 °C. In SGF, the actuator undergoes autonomous bending followed by shape recovery. In contrast, in HCl, only bending is observed without recovery.

**Video S2:** Four distinct BSA-PEGDA/PEGDA bilayer hydrogel actuators with different geometries were fabricated via DLP 3D printing. Their autonomous multi-stage shape morphing behaviors were recorded in SGF (pepsin 0.5 mg/mL, pH ~2) at 37 °C.

**Video S3:** A 3D-printed BSA-PEGDA bilayer hydrogel gripper autonomously grasps and releases an object in SGFs (pepsin 0.5, 1 and 2 mg/mL, pH ~2) and HCl (pH ~2) at 37 °C.

**Video S4:** DOX release behavior of 3D-printed cylindrical BSA-PEGDA-DOX (C*_DOX-gel_*~0.2 mg/mL) hydrogels in SGF (pepsin 1 mg/mL, pH ~2), HCl (pH ~2), and TRIS at 37 °C.

**Video S5:** Two distinct BSA-PEGDA-DOX (C*_DOX-gel_*~0.8 mg/mL) bilayer hydrogel actuators with different geometries were fabricated via 3D printing. Their drug release profiles and autonomous multi-stage shape morphing behaviors were observed in SGF (pepsin 0.5 mg/mL, pH ~2) at 37 °C.

# ****2 Methods****

**Preparation of Bioink and 3D Printing of Hydrogels**

BSA powder and PEGDA (700 Da) were dissolved in TRIS (20 mM Tris, 150 mM NaCl, pH ~7.4) to prepare a mixture with final concentrations of 2.5 mM BSA and 125 mM PEGDA. The mixture was stirred at room temperature (RT) using a magnetic stirrer for 15 hours to form a stable BSA-PEGDA composite. Separately, under light-protected conditions, LAP was dissolved in TRIS to prepare a 150 mM stock solution. Concurrently, PEGDA was dissolved in TRIS to form a 250 mM solution. Both solutions were stirred at RT for 20 minutes. All solutions were centrifuged at 6000 rpm for 10 minutes to remove air bubbles and stored at 4°C until use. Fresh BSA-PEGDA mixture was prepared for each experiment. Bioinks were formulated by mixing either the BSA-PEGDA mixture or the PEGDA solution with the LAP stock solution at a 4:1 volume ratio. This resulted in final concentrations of 2 mM BSA, 100 mM PEGDA, and 30 mM LAP for the BSA-PEGDA bioink, or 200 mM PEGDA and 30 mM LAP for the PEGDA bioink. Hydrogels were fabricated using a BIONOVA X DLP 3D Bioprinter in a layer-by-layer manner. The printing parameters were set as follows: layer thickness of 50 μm, light intensity of 75%, and exposure time of 10 seconds per layer under violet light (405 nm) for crosslinking. After printing, the hydrogels were washed three times with TRIS (10 minutes per wash) to remove residual unreacted components and stored at 4°C for subsequent experiments.

**Optimizing Bioink Printability through Tartrazine Concentration Adjustment in 3D Printing**

Tartrazine was dissolved in TRIS under light-protected conditions at RT to prepare stock solutions at concentrations of 2% and 5% (w/v). The solutions were mixed on a rotary mixer for 20 minutes and subsequently stored at 4°C until use. To determine the optimal tartrazine concentration in bioinks, a spiderweb structure was designed using SolidWorks as a test model. Different volumes of the 2% and 5% (w/v) tartrazine stock solution were added to two bioink formulations: (1) BSA-PEGDA bioink, where 0, 5, 10, 15, 20, 25 or 30 μL of the 2% (w/v) tartrazine stock solution was added per 600 μL of bioink; (2) PEGDA bioink, where 0, 5, 15, or 30 μL of the 2% (w/v) tartrazine stock solution, or 15 μL or 20 μL of the 5% (w/v) tartrazine stock solution, was added per 600 μL of bioink. The mixtures were stirred until homogeneous and subsequently centrifuged at 6000 rpm for 5 minutes to remove air bubbles before printing. After printing, the clarity of spiderweb-like structures at different tartrazine concentrations was compared to evaluate its effect. The printed results were also compared with the original digital models to assess printing accuracy. C60, stent and spiral shapes were further printed to demonstrate the high precision of the optimized bioink.

**Preparation of Simulated Gastric Fluid (Pepsin, pH ~2)**

A 12 M stock HCl solution was diluted with double-distilled (DD) H_2_O to 0.01 M (pH ~2). The solution was mixed on a rotary mixer for 10 minutes to ensure uniformity. At RT, an appropriate amount of pepsin (≥2500 units/mg) powder was dissolved in the HCl (pH ~2) to prepare a 2 mg/mL pepsin solution. The 2 mg/mL pepsin solution was then serially diluted with the HCl (pH ~2) to concentrations of 1 and 0.5 mg/mL. All SGF was freshly prepared before experiments to ensure consistent pepsin activity.

**Swelling Ratio Measurements**

Cylindrical BSA-PEGDA hydrogel samples, with a diameter of 7 mm and a height of 1 mm, were prepared via 3D printing. After reaching swelling equilibrium, the samples were immersed at 37°C in 3 mL of TRIS, HCl (pH ~2), and SGFs (pepsin 0.5, 1, and 2 mg/mL, pH ~2). At time points of 0, 0.5, 1, 2, 3, and 4 hours, the respective samples were removed, gently blotted with absorbent paper to remove surface moisture, and their initial wet weight ($W_{wet}$) was recorded. Subsequently, each sample was washed at RT with 15 mL of DD H_2_O by rotary stirring three times, with each wash lasting 10 minutes, to remove TRIS residuals. For fully degraded BSA-PEGDA hydrogels, washing was performed with gentle stirring, and the washing liquid was carefully aspirated to preserve sample integrity as much as possible. The samples were then frozen in liquid nitrogen for 5 minutes and dried in a freeze-dryer for 24 hours, after which their dry weight ($W_{dry}$) was measured. Each experiment was conducted in triplicate, and the results are presented as the mean ± standard deviation (SD). The swelling ratio (SR) of the hydrogels was calculated using the following equation (1):

$$\mathrm{SR}\left( \% \right)=\frac{\left（ W_{wet}-W_{dry} \right）}{W_{dry}}\times100$$

where $W_{\text{wet}}$represents the swollen mass of the hydrogel after immersion in simulated gastric fluid (SGF), HCl (pH ~2), or TRIS for a specific time $t$, and $W_{\text{dry}}$ denotes the corresponding dry mass obtained after freeze-drying.

**Mechanical Compression Testing**

Cylindrical BSA-PEGDA hydrogels with a diameter of 7 mm and a height of 1 mm were printed using the optimized bioink. After printing, the hydrogel samples were washed at RT with TRIS three times, with each wash lasting 10 minutes, to remove residual material. The washed samples were then stored at 4°C for 24 hours to achieve equilibration. Subsequently, the saturated hydrogel samples were immersed in 3 mL of TRIS (as a control), HCl (pH ~2), and SGFs (pepsin 0.5, 1, and 2 mg/mL, pH ~2). The samples were incubated in a 37°C incubator, and at time points of 0, 0.5, 1, 2, 3, and 4 hours, samples were removed for testing. After each test, the samples were returned to their original solutions for continued incubation to observe long-term effects. Compression tests were conducted at RT using an INSTRON mechanical testing machine equipped with a 5000 N load cell. The test parameters included a compression rate of 1 mm/min and compression to 20% of the sample’s initial height. Stress-strain curves obtained from the tests were used to calculate Young’s modulus, determined as the slope of the curve within the 0~10% strain range. Prior to each test, the sample diameter was measured using a vernier caliper, and sample parameters were adjusted based on diameter changes to ensure data accuracy. To ensure reliability, all tests were performed in triplicate, and the results are presented as the mean ± standard deviation (SD).

**ATR-FTIR**

Cylindrical BSA-PEGDA hydrogel samples, with a diameter of 7 mm and a height of 1 mm, were prepared via 3D printing. After reaching swelling equilibrium, the samples were immersed in 3 mL of TRIS and SGF (pepsin 1 mg/mL, pH ~2), and incubated at 37°C for 4 hours. Subsequently, the samples were washed three times with TRIS, each wash lasting 10 minutes, to remove residual pepsin. The samples were then analyzed using a Nicolet iS50 FTIR spectrometer in ATR mode with a Type II a diamond crystal. Spectra were collected with 16 scans at a resolution of 8 cm⁻¹. Prior to testing, the background was calibrated with TRIS. The spectral data were processed by Y-axis normalization, background subtraction, and baseline correction. Changes in the amide I and II band absorption peaks within the 1500~1700 cm⁻¹ range were analyzed using OMNIC FTIR software to assess the effects of pepsin treatment on the hydrogel structure.

**ANS Fluorescence Detection**

BSA-PEGDA hydrogel samples, processed and washed following the same steps as in the ATR-FTIR analysis. Then, ANS solution was added to achieve a final concentration of 30 μM, enabling ANS to serve as a fluorescent probe for detecting changes in protein structure. The samples were then incubated at RT with shaking at 100 RPM for 10 minutes to ensure sufficient binding of ANS to BSA. Subsequently, ANS fluorescence changes in the hydrogels were recorded using a Bio-Rad ChemiDoc fluorescence imaging system, with consistent light source wavelength and exposure time maintained to ensure data comparability and accuracy.

**SDS-PAGE**

Cylindrical BSA-PEGDA hydrogel samples, with a diameter of 7 mm and a height of 1 mm, were prepared via 3D printing. After reaching swelling equilibrium, the samples were immersed in 3 mL of SGFs (pepsin 0.5, 1, and 2 mg/mL, pH ~2). The samples were then incubated in a 37°C incubator with shaking at 40 rpm for 4 hours. At time points of 10, 20, 30 minutes, and 1, 2, 3, and 4 hours, 300 μL of the solution was withdrawn for SDS-PAGE gel loading, and an equal volume of fresh SGF at the corresponding concentration was immediately added to maintain consistent conditions. Additionally, the original BSA-PEGDA (2.5-125 mM) mixture diluted 100-fold with TRIS and SGF at corresponding concentrations were prepared as reference. To compare the degradation behavior of free BSA and BSA incorporated within the hydrogel network, pure BSA (2 mM) and BSA-PEGDA precursor solutions (2-100 mM) were prepared with identical concentrations and total volumes to those used in the hydrogel samples. Each solution (equal in volume to one hydrogel specimen) was added into 3 mL of SGFs (pepsin 0.5, 1, or 2 mg/mL, pH ~2) or TRIS as a control and incubated at 37 °C under gentle shaking (40 rpm) for 4 h. For BSA-containing groups, 200 μL aliquots were collected at 0.5 h and 4 h in SGF, and additionally at 2 h for the pure BSA group to capture intermediate degradation states. For PEGDA-only (100 mM) control groups, samples were collected at the beginning (0 h). To ensure comparability, corresponding SGF control solutions (without BSA protein) were prepared at the same pepsin concentrations (0.5, 1, and 2 mg/mL), incubated under identical conditions (37 °C, 40 rpm, 4 h), and sampled at 0, 0.5, and 4 h. All collected samples were stored at 4 °C prior to SDS-PAGE analysis. Subsequently, a 4× Laemmli Sample Buffer was mixed with 2-mercaptoethanol at a 9:1 volume ratio, then combined with the sample or reference solutions at a 1:3 volume ratio. The mixtures were heated at 95°C for 5 minutes to denature proteins, impart a negative charge, and provide dye tracking. Precast SDS-PAGE gels were soaked in running buffer, and 5 μL of protein molecular weight standard (ladder) and 30 μL of sample or reference mixture solution were loaded into the gel wells. Electrophoresis was initiated at 150 V for 5 minutes, then reduced to 100 V until protein bands separated clearly. The gel surface was washed with DD H_2_O, and the gel was transferred to a Bio-Rad ChemiDoc MP imaging system. In Stain-Free mode, UV light was used to activate the reaction between photoactive agents in the gel and tryptophan residues, with an exposure time of approximately 1 minutes to generate a fluorescent signal. Images were captured directly using the ChemiDoc system without additional staining.

**Cryo-SEM**

Cylindrical BSA-PEGDA hydrogel samples, with a diameter of 7 mm and a height of 1 mm, were prepared via 3D printing. After reaching swelling equilibrium, the samples were immersed in 3 mL of TRIS and SGF (pepsin 1 mg/mL, pH ~2). Additionally, PEGDA hydrogel samples were immersed in 3 mL of TRIS. All samples were incubated in a 37°C incubator for 4 hours. Post-incubation, the TRIS and SGF-treated samples were washed at RT with 15 mL of DD H_2_O three times, with each wash lasting 10 minutes. Subsequently, the samples were fixed between two 3 mm aluminum discs in a high-pressure freezer (EM ICE, Leica) and transferred under vacuum cryogenic conditions via a loading station (EM VCM, Leica) to a freeze-fracture system (EM ACE900, Leica). In a cryogenic environment, the samples were fractured using a cooled knife and etched at -100°C for 10 minutes to sublimate surface ice crystals. The samples were then coated with a 3 nm thick carbon layer by sputtering. Finally, imaging was performed at -120°C using a field-emission scanning electron microscope (Gemini SEM, Zeiss) equipped with a secondary electron detector to observe the microstructure. All measurements were conducted at the Ilse Katz Institute for Nanoscale Science and Technology, Ben-Gurion University of the Negev, Beer Sheva, Israel.

**Cell compatibility Characterization**

BSA-PEGDA (2-100 mM) and PEGDA (200 mM) hydrogels were fabricated via 3D printing (diameter: 5 mm, height: 1 mm, BSA mass: 0.00262 g) and rinsed three times with phosphate-buffered saline (PBS, 10 min each) to remove unreacted residues. Cytocompatibility was evaluated using the mucus-secreting human colorectal epithelial cell line HT29-MTX, originally derived from ECACC (Cat. No. 12040401) and obtained through Sigma-Aldrich, following the culture protocol^[1]^. HT29-MTX, a goblet-like subclone derived from the human colorectal adenocarcinoma cell line HT-29, were cultured in Dulbecco’s Modified Eagle Medium (DMEM) supplemented with 10% fetal bovine serum (FBS) and 1% penicillin-streptomycin and maintained at 37 °C in a humidified 5% CO₂ atmosphere. Cells were seeded in 12-well plates at a density of 1 × 10⁵ cells per well and allowed to adhere for 24 h before testing. Two experimental configurations were applied: (i) direct contact, where hydrogels were placed directly into wells containing adherent cells; and (ii) indirect contact, where samples were placed in cell culture inserts (4 μm pore size) allowing only soluble components to diffuse toward the cells. Each configuration included one control group (cells without materials) and two test groups (BSA-PEGDA and PEGDA hydrogels). Prior to testing, hydrogels were sterilized by UV irradiation for 20 min, immersed in 5 mL of 70% ethanol for an additional 20 min, and washed thoroughly with DMEM to remove residual ethanol. For the direct contact assay, 2 mL of culture medium was added to each well containing cells and test materials. For the indirect contact assay, inserts containing 1 mL of medium and hydrogel samples (donor chamber) were placed in wells containing 2 mL of medium and adherent cells (acceptor chamber). The culture medium was refreshed every 72 h. Cell morphology was examined using a bright-field microscope (20× magnification) on Day 7. On Day 7, cell viability was quantified using the MTT assay. After removing the medium, cells were rinsed twice with PBS, incubated with 1 mL of MTT solution (5 mg/mL) for 1 h at 37 °C, and the resulting formazan crystals were dissolved in 1 mL of dimethyl sulfoxide (DMSO). The absorbance was measured at 550 nm using a microplate spectrophotometer. Cell viability was expressed as a percentage relative to untreated control cells (defined as 100% viability), and calculated according to equation (2):

$$Viabilty \left( \% \right)=\frac{Absorbance of treated cells}{Absorbance of untreated control cells}*100$$

**Preparation of Pepsin and Pepstatin A mixture**

To prepare a pepstatin A (PepA) stock solution, an appropriate amount of PepA powder was weighed and dissolved in 96% (v/v) ethanol in a sealed container to prevent evaporation. The mixture was gently agitated on a heating block at 50°C for approximately 1 hour until fully dissolved, yielding a stock solution with a concentration of 2 mg/mL (2920 μM). The solution was aliquoted and stored at -20°C until use. For experimental use, the PepA stock solution was mixed with a freshly prepared SGF (pepsin 1 mg/mL, pH ~2) at RT for 15 minutes to prepare mixed solutions with C*_PepA_~*0, 15, 20, and 30 μM. During this process, the ethanol concentration was diluted to below 1% (v/v), which had no significant effect on BSA or pepsin activity^[2,3]^. The mixed solutions were prepared immediately before use in subsequent experiments.

**Bilayer Hydrogel Preparation and Shape morphing Analysis**

A rectangular model with dimensions of 9 mm × 3 mm × 0.6 mm was designed using SolidWorks and uploaded to a 3D printer for fabrication. Bilayer hydrogels were prepared with the first layer consisting of PEGDA hydrogel (200 mM) and the second layer of BSA-PEGDA hydrogel (2-100 mM), each layer having a thickness of 0.3 mm. After printing, the samples were washed three times with TRIS, each wash lasting 10 minutes, to remove unreacted components. Upon reaching swelling equilibrium, the samples were immersed in 3 mL of HCl (pH ~2), SGFs (pepsin 0.5, 1, and 2 mg/mL, pH ~2), and SGF (pepsin 1 mg/mL, pH ~2) mixed with PepA at concentrations of 0, 15, 20, and 30 μM. The samples were incubated in a 37°C incubator, and shape morphing was recorded at 0, 10, 20, and 40 minutes, and 1, 1.5, 3, and 4 hours. For the PepA-containing groups, additional time points at 6, 8, 10, and 24 hours were recorded. Bending angles (θ) were analyzed using ImageJ software to quantify the deviation from the initial straight shape, assessing the bending and recovery behavior in different solution environments. Additionally, in HCl (pH ~2) and SGF (pepsin 0.5 mg/mL, pH ~2), the shape morphing process of the bilayer hydrogels was recorded via video to observe dynamic response characteristics.

Real porcine gastric fluid (PGF) was obtained within 2 h postmortem from healthy adult pigs obtained from a Rambam Medical Center slaughterhouse. In compliance with animal protection regulations, the pigs were not fasted prior to collection. The gastric fluid was centrifuged at 6000 RPM for 10 minutes to remove food debris, and its initial pH was measured. The pH was then adjusted to ~2 using a 1 M HCl solution to achieve a final concentration of 0.01 M, followed by dilution with HCl (pH ~2) at ratios of 4X, and 9X. Subsequently, the bilayer hydrogels were immersed in the diluted PGF samples and incubated in a 37°C incubator for 6 hours. Morphological changes were recorded at 0, 10, 20, 40, 60, 90, 180, 240, 300, and 360 minutes, with bending angle changes analyzed using ImageJ software.

**Complex Actuator Shape Design, Preparation, and Shape morphing**

Multiple hydrogel shape models were designed using SolidWorks, including a three-layer ring structure, a pyramid-shaped final form, a bamboo-like structure, a box-like structure. The three-layer ring structure consisted of a bottom layer of BSA-PEGDA (2-100 mM), a middle layer of PEGDA (200 mM), and a top layer of BSA-PEGDA (2-100 mM), with a diameter of 8 mm and a height of 0.9 mm. The pyramid-shaped, bamboo-like, and box-like hydrogels featured a bottom layer of PEGDA (200 mM) and a top layer of BSA-PEGDA (2-100 mM) with varying strip designs (width of 0.5 mm), each with a diameter of 8 mm and a height of 0.6 mm. All models were fabricated via 3D printing and washed three times with TRIS, each wash lasting 10 minutes. After reaching swelling equilibrium, the three-layer ring, pyramid-shaped, bamboo-like, and box-like samples were immersed in 3 mL of SGF (pepsin 0.5 mg/mL, pH ~2), real porcine gastric fluid (4X dilution, pH ~2), and HCl (pH ~2) and incubated in at 37°C. Shape morphing was recorded at 0, 20, and 360 minutes, with the full morphing process in SGF (pepsin 0.5 mg/mL, pH ~2) captured on video.

**Functional Characterization of Soft Untethered Grippers**

To evaluate the autonomous grasping capability of the gripper, a four-petal gripper was printed, consisting of a bilayer structure with a bottom layer of PEGDA (200 mM) and a top layer of BSA-PEGDA (2-100 mM). The gripper had a diameter of 8 mm and a height of 0.6 mm, and included a closed ring structure for shape constraint during printing. For functional testing, the gripper was suspended from the top using a thin inert metal wire and immersed in equal volumes of HCl (pH ~2) and SGFs (pepsin 0.5, 1, and 2 mg/mL, pH ~2). The samples were incubated at 37 °C to mimic gastric conditions. A camera system was used to continuously record the shape morphing process, which included the gripper autonomously closing to grasp a plastic ball, lifting the ball, relocating it to a designated position and height, and finally releasing it to return to its original flat state.

To test the gripper's autonomous ability to catch and penetrate the mucus layer, the simulated gastric mucus layer was prepared following previously reported mucin-based protocols^[4]^. A four-armed therapy gripper was fabricated via 3D printing, with sharp structures designed at the end of each arm to enhance mucus layer gripping and penetration capabilities. To improve gripping stability, rigid microneedles with a height of 0.9 mm were printed at the end of each arm using 25% (w/v) PEGDA. The gripper was printed in three layers: a base layer of BSA-PEGDA, a middle layer of PEGDA (200 mM), and a final microneedle layer composed of 25% (w/v) PEGDA. Catching performance was tested under two fluid environments: HCl (pH ~2) and SGF (pepsin 1 mg/mL, pH ~2).

**Fluorescence Spectroscopy Analysis of BSA-PEGDA-DOX Complex**

To investigate the interaction between doxorubicin (DOX) and BSA, fluorescence spectroscopy was utilized for quantitative analysis. At RT, DOX solutions were prepared in TRIS at concentrations of 0.045, 0.09, 0.18, and 0.36 mg/mL. A BSA-PEGDA solution (2.5-125 mM) was diluted with TRIS to a BSA concentration of 12 µM (0.798 mg/mL). Subsequently, the DOX stock solution was mixed with the BSA-PEGDA solution at a 1:5 volume ratio, yielding final DOX concentrations of 0.0075, 0.015, 0.03, and 0.06 mg/mL, with a consistent BSA concentration of 0.665 mg/mL. The mixed solutions were incubated in a 37°C incubator under light-protected conditions with gentle rotation for 24 hours to ensure sufficient binding between DOX and BSA. Fluorescence measurements were conducted using a 3 mL quartz cuvette, with parameters set at an excitation wavelength of 280 nm and an emission wavelength range of 287~500 nm. Changes in fluorescence intensity at 347 nm (the characteristic tryptophan peak of BSA) were monitored to analyze binding interactions. The binding mechanism was evaluated using modified Stern-Volmer equation linear fitting (3), with the equation expressed as follows^[5]^:

$$\frac{F_{0}}{F_{0}-F}=\frac{1}{f}+\frac{1}{fK\left[ drug \right]}$$

Where $F_{0}$is the fluorescence intensity without drug, $F$ is the fluorescence intensity measured after drug addition, $K$is the binding constant, $\left[ drug \right]$is the drug concentration, and $f$represents the fraction of fluorophores that are accessible to the quencher, indicating the proportion of total fluorescence that can be affected by the interaction. The plot of $\frac{F_{0}}{\left( F_{0}-F \right)}vs.\frac{1}{\left[ drug \right]}$ was constructed, and the slope $\frac{1}{K}$ was used to calculate the binding constant$K$.

**Optimization of BSA-PEGDA-DOX Bioink and Synthesis of DOX-Containing Hydrogels**

Stable bioconjugate BSA-PEGDA complex solution (2.5-125 mM) and PEGDA solution (250 mM) were prepared as described previously and centrifuged at 6000 rpm for 10 minutes to remove air bubbles. DOX powder was added to the BSA-PEGDA solution to achieve final concentrations (C*_DOX-pre_*~0.25, 0.5, and 1 mg/mL), while the PEGDA solution was adjusted to a final concentration (C*_DOX-pre_*~1mg/mL). The solutions were incubated in a 37°C incubator under light-protected conditions with gentle rotary stirring for 24 hours to ensure complete dissolution of DOX and formation of BSA-PEGDA-DOX and PEGDA-DOX complex solutions. The solutions were then centrifuged again at 6000 rpm for 10 minutes to confirm the absence of DOX precipitates. Subsequently, these solutions were mixed with LAP (150 mM) at a 4:1 volume ratio to prepare BSA-PEGDA (2-100 mM)-DOX (C*_DOX-ink_*~0.2, 0.4, and 0.8 mg/mL), and PEGDA (200 mM)-DOX (C*_DOX-ink_*~0.8 mg/mL) bioinks. To enhance printing precision, given the slight absorption of DOX at 405 nm (C*_DOX-ink_*~0.8 mg/mL), the tartrazine (2%, w/v) volume ratio in the BSA-PEGDA-DOX bioink at this concentration was adjusted to 40:1, while lower DOX concentrations (C*_DOX-ink_*~0.2, and 0.4mg/mL) used a 30:1 ratio, and the PEGDA-DOX bioink used a 20:1 ratio. Then, BSA-PEGDA-DOX and PEGDA-DOX hydrogels were then fabricated via 3D printing. After printing, the samples were washed in a 37°C incubator with 15 mL of TRIS three times, each wash lasting 1 hour, to remove free DOX, and subsequently stored under light-protected conditions at 4°C for 24 hours to achieve swelling equilibrium.

**Circular Dichroism Spectroscopy**

BSA (2.5 mM) solution, BSA-PEGDA (2.5-125 mM) conjugate mixture, BSA (2.5 mM)-DOX (C*_DOX-pre_*~1mg/mL) and BSA-PEGDA (2.5-125 mM)-DOX (C*_DOX-pre_*~1 mg/mL) complexes were prepared as described above and subsequently diluted 600-fold (1 μL stock into 599 μL TRIS), and their CD spectra were recorded using a Chirascan Spectrometer in the far-UV region (190~260 nm). Measurements were performed in a 0.1 cm path-length quartz cell under a nitrogen atmosphere. BSA concentration was maintained at 4.17 μM, with DOX fixed at 3 μM. Spectra were averaged over three scans at a scan speed of 50 nm/min, with data collected at 1 nm intervals from 260 to 190 nm at 25°C. Buffer contributions were subtracted, and spectra were converted to mean residue ellipticity (MRE, deg·cm²·dmol⁻¹) using Standard Analysis software.

**Cumulative DOX Release Analysis**

UV-Vis spectroscopy was used to establish a DOX release calibration curve and perform quantitative analysis. Initially, DOX stock solutions (0.125 mg/mL) were prepared in TRIS and HCl (pH ~2), centrifuged at 6000 rpm for 5 minutes to confirm the absence of precipitates, and diluted at RT with the respective buffer in a 1:1 gradient to a minimum concentration of 0.01563 mg/mL. Additionally, a separate set of DOX gradient dilutions was prepared in HCl (pH ~2), supplemented with pepsin powder (final concentration 1 mg/mL), and incubated at 37°C for 10 minutes to ensure sufficient interaction. The resulting homogeneous solutions were analyzed by UV–Vis spectroscopy, monitoring absorbance at 480 nm. Baseline corrections were performed using the corresponding blank solutions (TRIS, HCl pH ~2, or pepsin 1 mg/mL in HCl pH ~2). Aliquots of 100 µL were transferred to a transparent 96-well plate, with each concentration measured in triplicate, and the average absorbance and SD were calculated to assess data stability. Calibration curves were constructed with DOX concentration (0~0.125 mg/mL) on the x-axis and absorbance on the y-axis, and linear regression equations and correlation coefficients (R²) were determined.

Subsequently, cylindrical BSA-PEGDA-DOX (C*_DOX-gel_*~0.2, 0.4, and 0.8 mg/mL) hydrogels and PEGDA-DOX hydrogels (C*_DOX-gel_*~0.8 mg/mL), each with a diameter of 7 mm and a height of 1 mm, were fabricated via 3D printing and immersed in 3 mL of TRIS, HCl (pH ~2), or SGF (pepsin 1 mg/mL, pH ~2). Additionally, a cylindrical BSA-PEGDA-DOX (C*_DOX-gel_*~0.8 mg/mL) hydrogel was placed in SGF (pepsin 1 mg/mL, pH ~2) containing varying PepA concentrations (C*_PepA_~*0, 15, and 30 μM). DOX release experiments were conducted in a 37°C incubator with gentle shaking at 40 rpm under light-protected conditions to simulate different physiological environments. At time points of 0, 0.5, 1, 2, 3, 4, 24, 48, and 72 hours, 100 µL of solution was withdrawn for absorbance measurement, and 100 µL of the corresponding fresh solution was added to maintain constant volume. The amount of DOX released into the 3 mL medium ($M_{t}$) at each time point was quantified using the respective calibrate curves. However, the DOX concentration measured in the different solutions were relatively low. Within this lower concentration range, the slopes of the linear calibration curves obtained in TRIS, HCl, and SGF were highly similar. To minimize analytical variability, the TRIS-based calibration curve$(y=4.2x, R^{2}=0.999)$ was consistently used for quantification. To determine the total DOX loading capacity $M_{0}$ of cylindrical BSA-PEGDA-DOX hydrogels, hydrogels containing different initial DOX concentrations (C*_DOX-gel_*~0.2, 0.4, and 0.8 mg/mL) were incubated in SGF (pepsin 1 mg/mL, pH ~2) under light-protected conditions with shaking at 40 rpm for 24 hours to ensure complete degradation (**see Supplementary Figure S6d**). After thorough mixing, 100 μL of the supernatant was collected from each sample for absorbance measurement. The initial DOX content was quantified using a pre-established calibration curve in TRIS. For PEGDA-DOX hydrogel, only the cumulative released mass (μg) was recorded. Three independent replicates were prepared for each sample, and the results are presented as the mean ± SD. The cumulative DOX release percentage (%) from BSA-PEGDA-DOX hydrogels was calculated using the following equation (4):

$$Cumulative Release \left( \% \right)=\frac{M_{t}}{M_{0}}*100$$

Additionally, DOX release from BSA-PEGDA-DOX (C*_DOX-gel_*~0.2 mg/mL) hydrogel was recorded via fluorescence video in TRIS, HCl (pH ~2), and SGF (pepsin 1 mg/mL, pH ~2) at 37°C over 4 hours.

The Korsmeyer-Peppas model was applied to the first 60% of the drug release data for linear regression analysis to establish a kinetic model, using the equation^[6]^ (5):

$$log\left( M_{t}/M_{0} \right)=logk+nlog\left( t \right)$$

Where $t$ is the time point, $k$is the release rate constant, and $n$ is the release exponent determining the release mechanism.

**DOX Release Kinetics of Bilayer Hydrogel Actuators**

Bilayer rectangular hydrogel actuators (9 mm × 3 mm × 0.6 mm) composed of BSA-PEGDA-DOX (C*_DOX-gel_*~0.8 mg/mL) and PEGDA were fabricated via 3D printing. After washing and reaching swelling equilibrium, the actuators were placed in 3 mL of TRIS, HCl (pH ~2), or SGF (pepsin 1 mg/mL, pH ~2). They were then incubated at 37 °C under light-protected conditions with gentle shaking at 40 rpm to promote shape morphing and drug release. Using the previously described method, shape morphing processes and DOX release masses were recorded at 0, 10, 20, 40, 60, 90, 180, and 240 minutes. The cumulative DOX release percentage and bending angle changes were calculated, while the initial DOX loading capacity ($M_{0}$) was determined based on the volume ratio of the BSA-PEGDA-DOX layer to the total cylindrical BSA-PEGDA-DOX hydrogel volume, using the equation (6):

$$M_{0}^{layer}=M_{0}^{cylindrical}*\left( \frac{V_{layer}}{V_{cylindrical}} \right)$$

Where $M_{0}^{layer}$ is the initial DOX loading in the BSA-PEGDA-DOX (C*_DOX-gel_*~0.8 mg/mL) hydrogel layer, $M_{0}^{cylinderical}$ is the total initial DOX loading in the entire cylindrical BSA-PEGDA-DOX (C*_DOX-gel_*~0.8 mg/mL) hydrogel, $V_{layer}$ is the volume of the BSA-PEGDA-DOX layer, $V_{cylindrical}$ is the total volume of the cylindrical hydrogel.

Additionally, BSA-PEGDA-DOX (C*_DOX-gel_*~0.8 mg/mL)/PEGDA hydrogels with pyramid and bamboo-like shapes were prepared and immersed in SGF (pepsin 0.5 mg/mL, pH ~2) and HCl (pH ~2). Shape morphing was observed, and photographs were taken at 0, 20, and 240 minutes. Concurrently, a camera was used to monitor the shape changes of these samples over 4 hours at 37°C.

**Statistical Analysis.**

All quantitative measurements were performed using n = 3 independent samples, and results are reported as mean ± standard deviation (SD). No additional data preprocessing, normalization, or statistical hypothesis testing was applied. Sample size (n) and data presentation format are also provided in each corresponding figure caption. Statistical analysis and plotting were performed in OriginLab software.

# ****References****

[1] J. C. Imperiale, I. Schlachet, M. Lewicki, A. Sosnik, M. M. Biglione, *Polymers* **2019**, *11*, 1862.

[2] R. Liu, P. Qin, L. Wang, X. Zhao, Y. Liu, X. Hao, *Journal of Biochemical and Molecular Toxicology* **2010**, *24*, 66.

[3] J. Puurunen, *Digestion* **1982**, *23*, 97.

[4] N. N. Porfiryeva, I. Zlotver, M. Davidovich-Pinhas, A. Sosnik, *Macromolecular Bioscience* **2024**, *24*, 2400028.

[5] D. Agudelo, P. Bourassa, J. Bruneau, G. Bérubé, É. Asselin, H. A. Tajmir-Riahi, *PLoS ONE* **2012**, *7*, e43814.

[6] R. W. Korsmeyer, R. Gurny, E. Doelker, P. Buri, N. A. Peppas, *International Journal of Pharmaceutics* **1983**, *15*, 25.
